# Microfabrication Technologies for Nanoinvasive and High‐Resolution Magnetic Neuromodulation

**DOI:** 10.1002/advs.202404254

**Published:** 2024-10-24

**Authors:** Changhao Ge, Tahereh Masalehdan, Mahdieh Shojaei Baghini, Vicente Duran Toro, Lorenzo Signorelli, Hannah Thomson, Danijela Gregurec, Hadi Heidari

**Affiliations:** ^1^ Microelectronics Lab (meLAB) James Watt School of Engineering University of Glasgow Glasgow G12 8QQ UK; ^2^ Biointerfaces lab, Faculty of Sciences Friedrich‐Alexander‐Universität Erlangen‐Nürnberg Henkestraße 91 91052 Erlangen Germany

**Keywords:** magnetic stimulation, microfabrication, nanoinvasive, neuromodulation, neurophysiology

## Abstract

The increasing demand for precise neuromodulation necessitates advancements in techniques to achieve higher spatial resolution. Magnetic stimulation, offering low signal attenuation and minimal tissue damage, plays a significant role in neuromodulation. Conventional transcranial magnetic stimulation (TMS), though noninvasive, lacks the spatial resolution and neuron selectivity required for spatially precise neuromodulation. To address these limitations, the next generation of magnetic neurostimulation technologies aims to achieve submillimeter‐resolution and selective neuromodulation with high temporal resolution. Invasive and nanoinvasive magnetic neurostimulation are two next‐generation approaches: invasive methods use implantable microcoils, while nanoinvasive methods use magnetic nanoparticles (MNPs) to achieve high spatial and temporal resolution of magnetic neuromodulation. This review will introduce the working principles, technical details, coil designs, and potential future developments of these approaches from an engineering perspective. Furthermore, the review will discuss state‐of‐the‐art microfabrication in depth due to its irreplaceable role in realizing next‐generation magnetic neuromodulation. In addition to reviewing magnetic neuromodulation, this review will cover through‐silicon vias (TSV), surface micromachining, photolithography, direct writing, and other fabrication technologies, supported by case studies, providing a framework for the integration of magnetic neuromodulation and microelectronics technologies.

## Graphical abstract

Advancing neuromodulation requires enhanced stimulation resolution. Magnetic stimulation, known for minimal signal loss and tissue damage, is pivotal but current methods like transcranial magnetic stimulation lack precise targeting. Future technologies must achieve submillimeter resolution and selective neuron modulation. This study examines invasive and nanoinvasive techniques, emphasizing microfabrication's crucial role in developing high‐resolution magnetic neurostimulation for research and clinical applications.

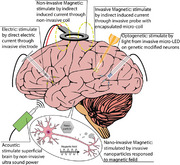

## 1 Introduction

Over centuries of observations, experimentation, and surgical exploration, researchers have established a profound understanding of the human brain and nervous system.^[^ ^1^ ^]^ Building on this progress and the ability to generate precisely controlled artificial electrical currents and fields, neuromodulation has undergone substantial optimization,^[^ ^2^, ^3^ ^]^ leading to its widespread adoption as a treatment for various neurological conditions, including headaches,^[^ ^4^ ^]^ Parkinson's disease,^[^ ^5^ ^]^ and epilepsy.^[^ ^6^ ^]^ The global burden of neurological disorders, measured by Disability‐Adjusted Life Years (DALYs), has risen from 6.29% in 2005 to 6.39% in 2015, with projections of further increase to 6.77% by 2030. Additionally, neurological disorders contribute to over 11% of global mortality annually.^[^ ^7^ ^]^ Consequently, developing and implementing effective neuromodulation techniques can significantly reduce the individual and societal burden of these conditions, playing a crucial role in the future of healthcare.

**Figure 1:**
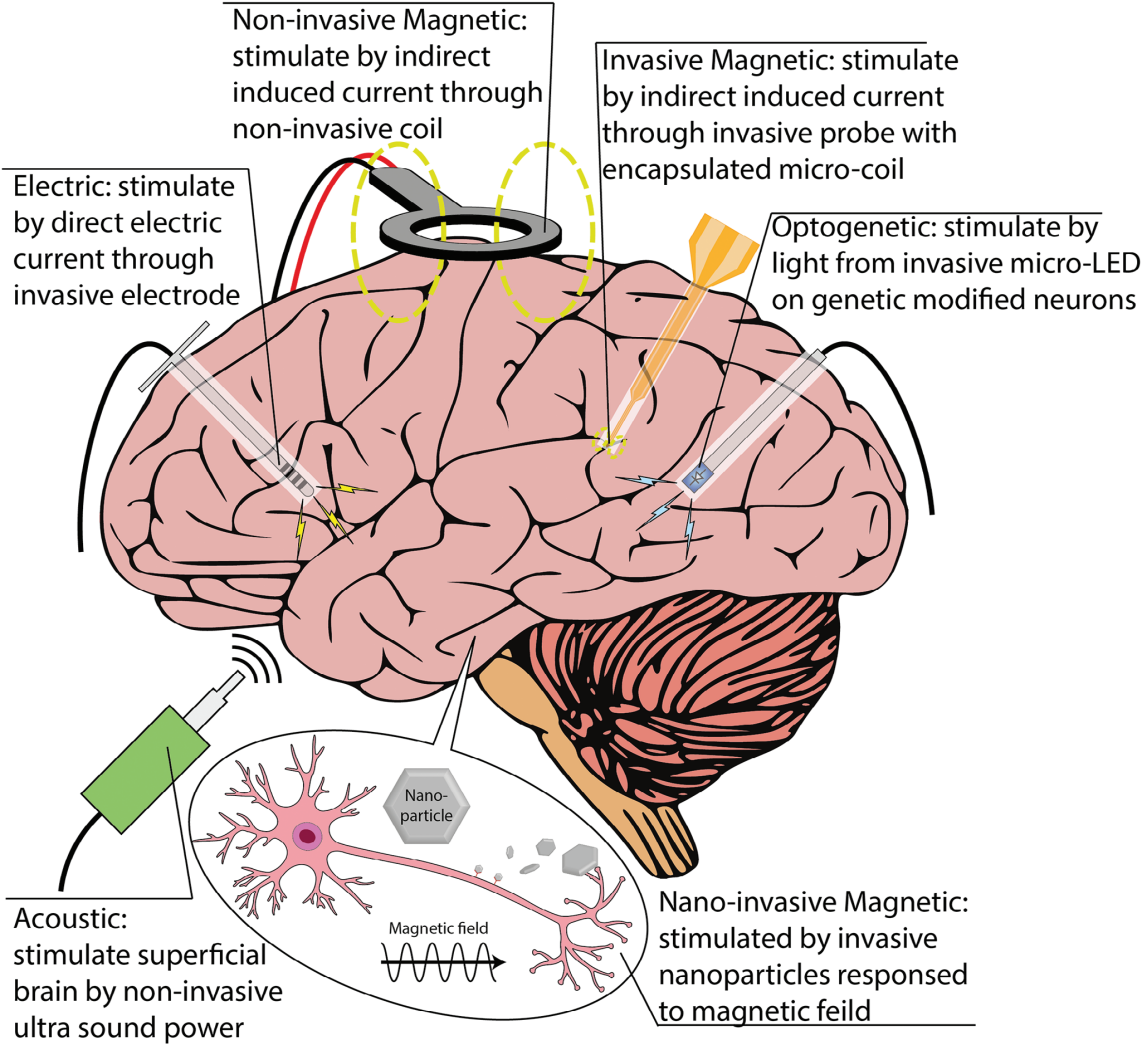
The schematic diagram illustrates various neuromodulation approaches for the brain, including electric, optogenetic, acoustic, noninvasive magnetic, invasive magnetic, and nanoinvasive magnetic neuromodulation.

Neuromodulation approaches can be systematically classified by the type of energy used to modulate neural activity, including electrical, magnetic, optical, and acoustic stimuli, as illustrated in **Figure** **1**. Electrical neuromodulation employs controlled fluctuations in voltage and current to modulate neuronal activity. While this approach is relatively mature, it is often associated with risks of pain and tissue injury, primarily due to invasive implantation procedures or direct scalp contact with large electrical stimulators.^[^ ^8^, ^9^ ^]^ Optical neuromodulation, commonly known as optogenetics, uses light to interact with genetically encoded photosensitive rhodopsin ion channels, achieving precise control of neuronal activity.^[^ ^10^ ^]^ Since these channels are nonmammalian, optogenetics typically requires genetic engineering of target neurons, which presents medical and ethical challenges.^[^ ^11^, ^12^, ^13^ ^]^ Transcranial ultrasound neuromodulation delivers ultrasonic waves to focal regions in the brain. It can be tuned to interact nondestructively with the membrane but faces the issue of signal absorption and loss.^[^ ^14^, ^15^ ^]^ In contrast, magnetic neuromodulation approaches, including noninvasive, invasive, and nanoinvasive magnetic neuromodulation, offer potential solutions to these limitations. Noninvasive magnetic methods cause significantly less pain than their electric counterparts.^[^ ^16^, ^17^, ^18^ ^]^ Invasive magnetic methods rely on microelectronic implants, offering high spatial resolution without genetic modification and causing fewer side effects than invasive electric methods.^[^ ^19^ ^]^ Nanoinvasive approaches use nanomaterials, and although genetic modification is often required, these methods provide highly selective modulation with minimal invasiveness. Additionally, a common advantage for all approaches is that low‐radiofrequency magnetic fields have a low absorption rate by human tissue.^[^ ^20^ ^]^ However, this requires careful coil design to control and steer the magnetic field effectively. With advances in microfabrication technology, miniaturization of large stimulation devices is becoming more feasible. Mini and microcoils offer flexible magnetic field sources, supporting accurate magnetic neuromodulation. Their small, flexible geometry makes them suitable for wearable and implantable applications, contributing to the future of mobile healthcare.

This review focuses on the development of magnetic neuromodulation techniques and related microfabrication technologies for mini/microcoil fabrication. Noninvasive neuromodulation includes Transcranial Magnetic Stimulation (TMS), which uses a time‐varying magnetic field from an external coil to stimulate neurons. Invasive techniques use micromagnetic stimulation (µMS), where neurons are stimulated by implantable microcoils. Nanoinvasive neuromodulation is achieved through implantable magnetic nanoparticles (MNPs), controlled by magnetic fields generated from external coils. The development and principles of each method are presented in Sections 2 and 3. Following this, Section 4 introduces multiple 2D and 3D microfabrication methods, including case studies on mini/microcoil development. Finally, Section 5 discusses the potential future applications of each technology and strategies for improvement.

## 2 Magnetic Neuromodulation Technologies

### 2.1 Noninvasive Neuromodulation

Noninvasive magnetic neuromodulation uses magnetic fields to induce current within tissues, enabling electrical neuromodulation. The earliest recorded experiment involving noninvasive magnetic neuromodulation dates back to 1896, when human patients received cortical stimulation through a large circular coil placed around the head, causing phosphenes, vertigo, and syncope.^[^ ^21^, ^22^ ^]^ It was not until the 1980s that researchers began performing stimulation of the peripheral nerve,^[^ ^23^ ^]^ and then the human motor cortex,^[^ ^24^ ^]^ naming the technique Transcranial Magnetic Stimulation (TMS). TMS, an almost painless alternative, competes with transcranial electrical stimulation in both research and therapeutic applications.^[^ ^24^, ^25^, ^26^, ^27^ ^]^ Following the initial clinical assessment,^[^ ^28^ ^]^ TMS rapidly gained recognition as a method applicable in both research and clinical contexts by the end of the 1980s.

Today, TMS remains a key tool in the treatment of depression,^[^ ^29^ ^]^ pain,^[^ ^30^ ^]^ seizure,^[^ ^31^ ^]^ and various other neurological disorders.^[^ ^32^, ^33^ ^]^ However, even with mapping, high‐resolution TMS can only achieve spatial accuracy on the centimeter scale. This resolution decreases with penetration depth, limiting its applicability in deep brain regions.^[^ ^34^, ^35^ ^]^

### 2.2 Invasive Magnetic Neuromodulation

Invasive magnetic neuromodulation was developed to overcome the limitations of noninvasive techniques, particularly in targeting specific, small brain regions. It involves implanting sub‐millimeter coils, known as microcoils, directly into the target tissue, significantly enhancing the resolution of stimulation. As a result, invasive magnetic neuromodulation is often referred to as micromagnetic Stimulation (µMS).

The development of µMS began in 2010 with the design and evaluation of millimeter‐scale mini‐coils that successfully induced neural responses in primates.^[^ ^36^ ^]^ In 2012, coil dimensions were reduced to a sub‐millimeter scale, demonstrating their effectiveness in exciting neurons in the retina in vitro.^[^ ^37^ ^]^ By 2013, further design improvements allowed researchers to investigate the efficacy of µMS in modulating the cochlear nucleus–inferior colliculus auditory pathway at the systems level.^[^ ^38^ ^]^ In 2014, studies showed that µMS could suppress subthalamic nucleus activity by 70%, a result comparable to traditional electrical neuromodulation methods.^[^ ^39^ ^]^ Both computational simulations and experimental studies in subsequent years have validated the potential of µMS in neuromodulation, significantly improving the spatial resolution of magnetic stimulation.^[^ ^35^, ^40^, ^41^ ^]^ However, as an invasive method, like certain electrical stimulation approaches, it carries inherent risks associated with implantation surgeries and potential side effects.^[^ ^42^ ^]^

### 2.3 Nanoinvasive Magnetic Neuromodulation

Nanoinvasive magnetic neuromodulation is mediated by magnetic nanoparticles (MNPs) composed of ferromagnetic or superparamagnetic materials, typically ranging from a few nanometers to several hundred nanometers.^[^ ^43^, ^44^, ^45^ ^]^ The interest in applying MNPs in biomedicine is driven by two main advantages: their small size allows direct interaction with membranes or proteins such as ion channels, and their magnetic properties enable remote control at any tissue depth using external magnetic fields.^[^ ^46^, ^47^ ^]^ MNPs have been used in drug delivery, hyperthermia, and MRI imaging since the last century, and around 2010, they began to be applied in neuromodulation.^[^ ^48^, ^49^, ^50^ ^]^

Different MNPs respond differently to external magnetic fields, enabling magnetothermal or magnetomechanical neuromodulation. Isotropic MNPs generate mechanical stimuli that activate mechanosensory ion channels through ‘pull’ forces, though their use in vivo is limited by the need for a strong magnetic field gradient.^[^ ^51^ ^]^ Anisotropic MNPs, due to their vortex spins, generate torques when aligned with external alternating fields of a few Hz with tens of mT strength. In this magnetomechanical neuromodulation approach, the torque generated in the MNPs translates to stimuli for the activation of mechanosensitive ion channels such as PIEZO1 and transient receptor potential cation channel subfamily V member 4 (TRPV‐4).^[^ ^52^, ^53^, ^54^ ^]^ When external magnetic fields oscillate at frequencies of several hundred kHz, MNPs with high magnetic saturation can generate heat, a process known as magnetic hyperthermia, which is used in magnetothermal neuromodulation targeting thermosensitive ion channels like transient receptor potential subfamily V member 1 (TRPV‐1).^[^ ^49^, ^55^, ^56^ ^]^

Increasing research demonstrates the ability of nanoinvasive methods to target and activate neurons with high spatiotemporal resolution,^[^ ^20^, ^57^, ^58^, ^59^, ^60^, ^61^, ^62^ ^]^ fueling advancements in technology and their application to brain disorders. However, some of these approaches often require genetic modification of neurons to express receptors sensitive to pressure or temperature, which are not naturally prevalent in the central nervous system.

**Figure 2:**
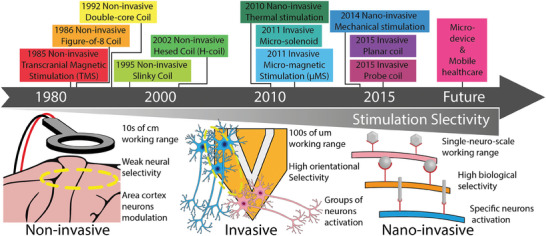
Upper half: A brief timeline of the starting point for magnetic neuromodulations and their related technologies. Lower half: Selectivity comparison between each type of magnetic neuromodulation.

A timeline of the landmark development and a comparison of characteristics for the three magnetic stimulation are shown in **Figure** **2**.

## 3 Principles of Magnetic Neuromodulation

The intricate process of communication between cells in the human body involves a dynamic interplay of chemical, mechanical, and electrical signals.^[^ ^63^ ^]^ Electrical signals arise from dynamic changes in the distribution of ions–such as sodium, potassium, calcium, and chloride–across cell membranes, facilitated by specialized ion channels and pumps. This process is a fundamental mechanism for cellular communication in neurons. Magnetic neuromodulation works by regulating the transmembrane transport of ions, either directly or indirectly, through magnetic fields to promote or inhibit neural excitation.

### 3.1 Noninvasive Magnetic Neuromodulation

TMS, as the first generation of magnetic stimulation, plays a significant role in the development of magnetic neuromodulation due to its ability to noninvasively modulate neuronal activity. This section introduces the operational principles of TMS and outlines subsequent improvements in its application.

#### 3.1.1 Mathematical and Physical Theory

The operational foundation of TMS is based on Faraday's law of electromagnetic induction, where a time‐varying magnetic field (B‐field) induces an electric field (E‐field) within cerebral tissue.^[^ ^64^, ^65^, ^66^, ^67^ ^]^ The induced electric field modulates transmembrane ion flow, leading to changes in neuronal membrane potential, which either depolarizes the neuron, increasing excitability or hyperpolarizes it, reducing excitability.^[^ ^68^ ^]^ This induced electric field is instrumental in provoking neuronal activity and responses. The mathematical relationship for a sinusoidal magnetic field is described by the differential form of Maxwell's equations as follows

(1) $$\nabla \times \vec{E}=-j\omega \vec{B}$$

(2) $$\nabla \times \vec{H}=\vec{J}+j\omega \vec{D}$$

Here, $\vec{E}$ is electric field intensity, $\vec{D}$ is electric displacement vector, $\vec{H}$ is magnetic field intensity and $\vec{B}$ is magnetic flux density. They are correlated as $\vec{D}=\varepsilon \vec{E}$ and $\vec{B}=\mu \vec{H}$, where ε and µ are permittivity and permeability, respectively. The electric charge density and electric current density are denoted by ρ and $\vec{J}$, respectively. The angular velocity of the sinusoidal field is denoted by ω and is directly proportional to the frequency *f* of the signal, where ω  =  2π*f*. Given that the presence of free charge within brain tissue can be considered negligible,^[^ ^19^, ^41^ ^]^ we get

(3) $$\vec{E}=-j\omega \vec{A}$$

Here, the magnetic vector potential, *A*, can be calculated by

(4) $$\vec{A}=\frac{\mu }{4\pi }\int_{V}\frac{\vec{J}}{R}dv$$

While the induced electric field can be expressed as

(5) $$\vec{E}=-j\omega \frac{\mu }{4\pi }\int_{V}\frac{\vec{J}}{R}dv$$

In the case of a current carrying coil composed of *N* turns wire, Equation (5) can be transformed to

(6) $$\vec{E}=-j\mu fNi\int_{L}\frac{1}{2R}d\vec{l}$$

Here, *N* is the number of turns in the coil; *i* is the magnitude of the input current; $d\vec{l}$ is the differential vector in the same direction as the current unit vector in a turn of wire, *L* is the length of integrating route for one turn, *R* is the distance between $d\vec{l}$ and the point of interest. Since the parameters are known, Equation (6) can be utilized to calculate the electric field.

#### 3.1.2 Realization and Improvement

**Figure 3:**
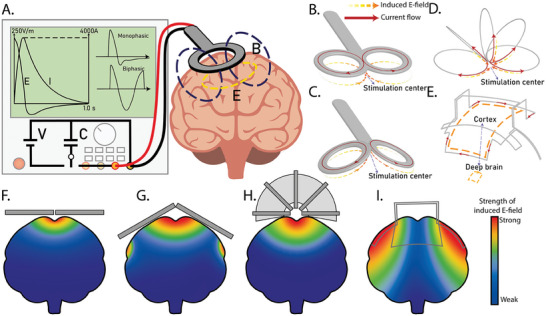
A) Working principle of first‐generation TMS with representative circuit diagram, example waveform of the current and induced electric field, and example monophasic and biphasic waveforms. B–I) Schematic diagrams of the coil and possible E‐field strength distribution in the brain cross‐section. In B–E) red arrows represent current flow; orange arrows represent induced electric field, deeper color means stronger field; in F–I) red transitioning to blue means strong transitioning to weak: B) “Figure‐of‐8” coil with stimulation focus at center, C) Double‐core coil with stimulation focus at center, D) Slinky coil (without core) with strong stimulation focus at center converge area, and E) simplified Hesed coil/H‐coil with relative small reduce of field strength between cortex and deep brain region, F) “Figure‐of‐8” coil centered field distribution, G) Double‐core coil centered deeper field distribution, H) Slinky coil (with core) centered deep field distribution, I) simplified Hesed coil/H‐coil decentralized low‐attenuation deep field distribution.

A standard TMS system consists of two main components: a stimulator and either a single coil or an array of coils. Early noninvasive neuromodulation systems used monophasic pulsed stimulation paradigms.^[^ ^24^ ^]^ The circuit that generates this pulsed signal connects a circular coil to a high‐voltage source and a large capacitor, as shown in **Figure** **3**. A. First, the capacitor is charged by the high‐voltage source. When the capacitor discharges, it releases a high current, reaching peak values of thousands of amperes within microseconds.^[^ ^16^, ^69^ ^]^ This current generates a magnetic field pulse strong enough to induce neuron depolarization.

During the early stages, TMS faced challenges in improving both stimulation effectiveness and spatial resolution. The issue of effectiveness was mainly addressed through improvements in stimulation signals. Early TMS techniques operated at low stimulation rates, with some delivering fewer than one pulse every 3 s.^[^ ^24^ ^]^ In response, the stimulation rate was increased, leading to the development of ‘rapid’ or ‘repetitive’ transcranial magnetic stimulation (rTMS), which is defined as TMS involving pulse trains at frequencies higher than 1 Hz.^[^ ^70^ ^]^ Initially, there were concerns about rTMS, particularly regarding the risks of tissue overheating^[^ ^71^, ^72^ ^]^ and the potential for seizures,^[^ ^73^ ^]^ despite supportive research.^[^ ^74^, ^75^ ^]^ With proper safety protocols, however, rTMS has become a widely accepted and popular stimulation method,^[^ ^76^, ^77^ ^]^ actively used in the treatment of various disorders, including pain management,^[^ ^78^ ^]^ movement disorder,^[^ ^79^ ^]^ and stroke rehabilitation.^[^ ^80^ ^]^ In addition to stimulation frequency, the shape of the pulse waveform is also an important factor. Monophasic and biphasic pulse shapes offer greater adaptability in clinical applications. For example, monophasic pulses induce stronger motor‐evoked potentials (MEP), while biphasic pulses produce shorter MEP latency and higher power efficiency.^[^ ^81^, ^82^, ^83^ ^]^

Efforts to enhance the spatial precision or focality of TMS have primarily focused on improving coil designs. Early generations of TMS coils featured a simple flat circular shape with a diameter of ≈10 cm, as illustrated in Figure 3A.^[^ ^24^ ^]^ However, this design did not provide the desired level of focal neuromodulation. Research showed that changing the coil size did not significantly improve TMS focality or efficacy.^[^ ^84^ ^]^ As a result, attention shifted to more innovative coil designs. In recent decades, various coil designs have been developed to address the challenge of enhancing stimulation focality. The following sections summarize important designs and their operating principles.

##### “Figure‐of‐8” Coil

This design consists of two flat circular coils positioned closely in the same plane, forming a figure “8” (as shown in Figure 3B).^[^ ^84^ ^]^ The critical aspect of this design is that the input currents flow in the same direction at the center of the “8,” where the coils intersect. As explained earlier, this arrangement intensifies the magnetic field at the intersection, as shown in Figure 3F.

In 1988, finite element calculations and in vivo frog nerve‐muscle experiments were conducted to test the feasibility of achieving “localized stimulation” using this coil design. The results showed that the target area had a current density 2 to 3 times higher than surrounding regions, confirming the potential of this coil design to enhance stimulation performance.^[^ ^85^ ^]^ Similar studies further validated the capabilities of this coil in generating stronger and more focused stimuli.^[^ ^84^, ^86^, ^87^, ^88^ ^]^ The “figure‐of‐8” coil provided significantly improved focal stimulation compared to the original TMS coil and has remained the most widely adopted design, influencing the mini/microcoil designs in next‐generation magnetic neuromodulation.

However, the “figure‐of‐8” coil has limitations. The stimulation strength must remain within an appropriate range, typically determined by the motor threshold, to avoid under‐ or overstimulation, as overstimulation can lead to side effects such as seizures.^[^ ^89^ ^]^ As explained by Equation (6), stimulation strength decreases with distance, making it difficult to generate safe fields for both cortical and deep brain stimulation (DBS) simultaneously.

##### Double‐Core Coil

The double‐core coil is a modification of the “figure‐of‐8” design, incorporating an angle between the two circular coils, as shown in Figure 3C.^[^ ^90^ ^]^ Recent research supports the double‐core coil's effectiveness in stimulating deep brain regions more efficiently than the “figure‐of‐8” coil, as shown in Figure 3. F. and H. As a result, the double‐core coil is gaining traction in the treatment of various conditions, including depression,^[^ ^91^ ^]^ and tinnitus.^[^ ^92^, ^93^ ^]^

##### Slinky Coil

The slinky coil consists of multiple circular coils evenly distributed along a path, rotating 180 degrees around one of its tangents, as shown in Figure 3D. This design converges current at the focal point, strengthening stimulation at the target area while minimizing field effects in other regions. The slinky coil can also be paired with a core (Figure 3H) to concentrate magnetic fields, increasing field density and improving efficiency compared to the standard “figure‐of‐8” coil. The slinky coil enhances focality and stimulation strength, offering a potential solution for DBS.^[^ ^94^, ^95^, ^96^, ^97^, ^98^ ^]^ However, like the “figure‐of‐8” coil, the slinky coil may lead to overstimulation due to high current density exceeding the motor threshold, posing a challenge for DBS that remains unresolved.

##### Hesed Coil (H‐Coil)

The Hesed coil, also known as the H‐coil, has a complex shape for DBS, as depicted in the simplified model in Figure 3E.^[^ ^98^ ^]^ Unlike other coils, the H‐coil spreads currents across the entire surface of the head rather than concentrating them on a single point. The additive effect of the distributed currents allows the H‐coil to effectively stimulate deep brain regions (Figure 3I) with a lower risk of overstimulating the cortex. Updated versions of the H‐coil simplified the wire layout, promoting a more uniform current distribution with just one port each for current input and output.^[^ ^99^, ^100^ ^]^ Studies have shown that the stimulation depth can reach 5–6 cm.^[^ ^100^ ^]^ Today, the H‐coil has gained popularity as a method for magnetic DBS.^[^ ^101^, ^102^, ^103^ ^]^ However, the H‐coil has drawbacks, including low selectivity, coil complexity, and its relatively large size, which may be burdensome for patients.

##### Alternative Designs

In addition to the above‐mentioned coil configurations, several alternative designs are worth considering. The “Halo coil” is a large circular coil that surrounds the cranial region and is generally used alongside other coils, such as the H‐coil and “figure‐of‐8” coil, to enhance DBS.^[^ ^104^, ^105^ ^]^ Another innovative design is the coil array,^[^ ^106^ ^]^ which consists of multiple coils arranged in sequence, each controlled independently. Simulations suggest that coil arrays may allow precise targeting without moving the coil, while also improving power management.^[^ ^107^ ^]^ Additionally, combining coils like the “figure‐of‐8” may enhance stimulation flexibility, strength,^[^ ^108^ ^]^ and penetration.^[^ ^109^ ^]^

### 3.2 Invasive Magnetic Neuromodulation

As discussed in Section 2, invasive magnetic stimulation is a follow‐on of TMS, sharing a common working principle to excite induced currents within tissues, thereby affecting neuromodulation. However, µMS, as an invasive modality, differs significantly from TMS in several key aspects.

Invasive magnetic neuromodulation requires coils small enough for implantation, making them much smaller than those used in traditional TMS. Because of their compact size, these coils must operate close to the target neurons. As a result, the spatial distribution and direction of the magnetic field are crucial, necessitating diverse coil designs for different applications. The following subsections will explore the operational mechanisms of invasive magnetic neuromodulation and the coil designs tailored to this approach.

#### 3.2.1 Working Principle

**Figure 4:**
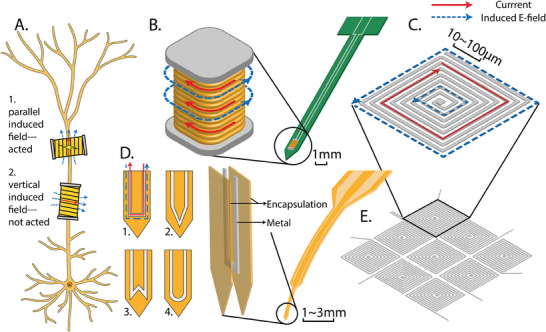
A) The orientational stimulation of neurons. The upper coil's electric field is parallel to the axon, the lower coil's is vertical. Only the upper coil successfully stimulates. B) The implantable microsolenoid on a PCB and a zoom‐in figure shows its current and field feature of the microsolenoid. C) A square planar coil with current and field features. D) An example probe coil with a zoom‐in structure diagram and 4 example designs of coil's shapes. E) An example coil array with 3 × 3 square planar coils.

Miniaturizing the coils to a sub‐millimeter scale offers several advantages, particularly enhanced stimulation selectivity. Research in the 20th century revealed that parallel electric field gradients applied to axons have a greater excitatory effect on neurons than electric fields applied vertically.^[^ ^110^, ^111^ ^]^ These findings highlight the potential for selective magnetic neuromodulation, a concept partially validated in TMS studies, although TMS's low resolution limits its application.^[^ ^90^, ^112^, ^113^ ^]^ However, with microcoils, µMS can fully realize this selectivity. Microsolenoids can strongly activate neurons in directions parallel to the coil wire but not in transverse directions.^[^ ^37^, ^39^, ^114^, ^115^ ^]^ As discussed in Section 3.1.1, the induced electric field is expected to align predominantly with the direction of current flow in the microcoil. Therefore, the positioning and shape of the microcoil significantly influence the stimulation performance of µMS, as depicted in **Figure** **4**A. In addition to selectivity, the constrained field distribution increases spatial resolution. Compared to the over 1 mm activation region typical of conventional invasive electric stimulation, µMS's stimulation is typically confined to a ≈300 µm diameter around the stimulation site.^[^ ^19^, ^116^ ^]^

#### 3.2.2 Coil Design

The coil is the cornerstone of µMS, and its design plays a crucial role in determining the selectivity of stimulation. Following is a summary of microcoil design and development.

##### Microsolenoid

Miniaturizing existing coil designs to the sub‐millimeter scale is a natural direction in µMS research. Among various coil types, the solenoid is one of the most commonly used and serves as an ideal reference for microcoil design. Figure 4B. provides an overview of the current and field directions. As a scaled‐down version, the microsolenoid retains a clear field map similar to its larger counterpart, making it easier to apply in µMS.

Early research consistently chose microsolenoids as the preferred design for microcoils.^[^ ^36^, ^37^, ^38^, ^115^, ^117^ ^]^ The first case of microcoil magnetic neuromodulation was demonstrated in 2012,^[^ ^36^ ^]^ where microcoils generated robust stimulation signals with spatial selectivity to activate retinal neurons. Subsequent research extended µMS applications to neurons in the brain.^[^ ^38^, ^117^ ^]^ In 2014, in vitro experiments on the mouse subthalamic nucleus demonstrated the effectiveness of inhibition through µMS.^[^ ^39^ ^]^ Later advancements included magnetic cores that produced stronger magnetic fields.^[^ ^118^, ^119^ ^]^ Recent studies have also used microsolenoids in configurations like the “figure‐of‐8” coil in TMS to enhance focality.^[^ ^120^ ^]^ There are also other productive works using this design including mouse in vivo neuron activation,^[^ ^121^, ^122^ ^]^ selectivity control,^[^ ^114^, ^123^ ^]^ and heat dissipation control,^[^ ^124^, ^125^ ^]^ among various other facets, solidifying the microsolenoid as the mainstream design in µMS.

##### Planar Coil

A planar coil is a coil arranged flat within a single plane. It typically uses a spiral wire layout to create multiple turns within that plane. An example of a square planar coil is shown in Figure 4C. Due to its flat structure, planar coils are better suited for under‐skull implants than for deep brain stimulation.

In 2015, a set of circular spiral coils with diameters ranging from 8 to 30 mm was analyzed for potential use as under‐skull implantable coils for TMS. The results showed that heat from the coil is manageable, but careful design of the coil's outer diameter is crucial, as a larger diameter allows for better heat dissipation.^[^ ^89^ ^]^ Since millimeter‐scale coils are not suitable for µMS, a square 50 µm‐by‐50 µm microplanar coil design was developed and analyzed for its ability to excite a 70 µm axon through simulation.^[^ ^126^, ^127^ ^]^ This square design has been refined in subsequent studies,^[^ ^128^, ^129^, ^130^, ^131^ ^]^ consistently remaining at a sub‐millimeter scale. This innovation is important for implant applications and has contributed to the successful execution of in vivo experiments on mice.^[^ ^132^ ^]^ Recently, in 2021, the square planar coil was integrated into a “figure‐of‐8” configuration to improve its focality.^[^ ^133^, ^134^ ^]^

##### Probe Coil

Probe coils are µm‐scale coils integrated into implanted prosthetic devices. These coils are often encased in a biocompatible matrix to enhance flexibility (Figure 4D).^[^ ^19^ ^]^ Probe coils feature intricate shapes to generate diverse electric field distributions, improving selectivity.^[^ ^19^, ^41^, ^135^ ^]^ A prototype probe coil demonstrated its selectivity potential based on previous theory.^[^ ^41^ ^]^ Simulations and experiments showed that adding an oblique segment intensified asymmetry between two orthogonal directions, improving selectivity.^[^ ^19^ ^]^

In 2020, semi‐circular probe coils successfully stimulated the mouse auditory cortex, showing a more constrained stimulation region compared to electric methods.^[^ ^116^ ^]^ In 2021, a probe coil configuration with a programmable circuit was introduced, allowing dynamic spatial programming to alter the stimulation focus.^[^ ^136^ ^]^ More recently, the propagation of neuromodulation through visual cortex layers was achieved, demonstrating the potential for stimulating complex neuronal activity.^[^ ^137^ ^]^ Additional research has examined material effects on probe coils^[^ ^138^ ^]^ and investigations into thermal effects.^[^ ^139^ ^]^ However, the principles of coil‐shape design are still being explored through a trial‐and‐error process.

##### Microcoil Array (Multi‐Micro‐Coil)

A microcoil array involves connecting multiple coils to perform specific functions.^[^ ^140^ ^]^ Examples include planar coil arrays (Figure 4.E),^[^ ^126^, ^127^, ^141^ ^]^ and microsolenoid arrays.^[^ ^142^, ^143^ ^]^ These arrays are designed to deliver precise modulation by activating the coil closest to the target neuron or enabling multi‐region modulation by using several coils simultaneously. Some microsolenoids and planar coils are also arranged in a “figure‐of‐8” coil style to enhance focality.^[^ ^120^, ^133^ ^]^ Some arrays incorporate probe coils to strengthen or weaken the field in a region of interest through currents flowing in the same or opposite directions.^[^ ^144^, ^145^, ^146^ ^]^ Independently controlling each coil in the array also enables more efficient energy management, a key factor in chronic modulation, which is one of the bases for chronic modulation.

As a summary, **Table** **1** lists key literature about applications of the coil designs discussed above.

**Table 1:** Publications overtime on µMS coil design.

Year	Contribution	In vivo/in vitro	Coil type	Refs.
2012	Demonstration of neuron activation via coils,	in vitro	Microsolenoid	[147]
2013	Demonstration of system‐level dorsal cochlear nucleus‐inferior colliculus neurons activation.	In vivo	Microsolenoid	[38]
2014	Adoption of microsolenoid array	–	Microsolenoid (array)	[140]
2014	Demonstration of cellular inhibition via micromagnetic stimulation	In vitro	Microsolenoid	[39]
2014	Demonstration of the role of field direction and single/repetitive stimulation in micromagnetic stimulation	In vitro	Microsolenoid	[148]
2016	Demonstration of feasibility of high selectivity microcoil	In vitro	Probe coil	[41]
2016	Adoption of planar coil and planar coil array	–	Planar coil (array)	[126]
2017	Animal microcoil implantation test	In vivo	Microsolenoid	[121]
2018	Adoption of selective probe coil designs	In vitro	Probe coil	[19]
2019	Animal microcoil array test	In vivo	Microsolenoid (array)	[142]
2020	Adoption of probe coil array	In vitro	Probe coil (array)	[144]
2023	Adoption of “figure‐of‐8” microsolenoid	–	Microsolenoid (array)	[120]

### 3.3 Nanoinvasive Magnetic Neuromodulation

Nanoinvasive magnetic neuromodulation differs significantly from other magnetic neuromodulation techniques. While TMS and µMS rely on electromagnetic induction for stimulation, nanoinvasive magnetic stimulation uses implantable nanoparticles to modulate neuronal activity. The more complex modulation principles in nanoinvasive stimulation mean that various factors, such as nanoparticle materials, size, shape, ion channel targeting, and coil configuration, significantly impact performance. Therefore, a comprehensive understanding of how these components interact is crucial.

#### 3.3.1 Working Principles

**Figure 5:**
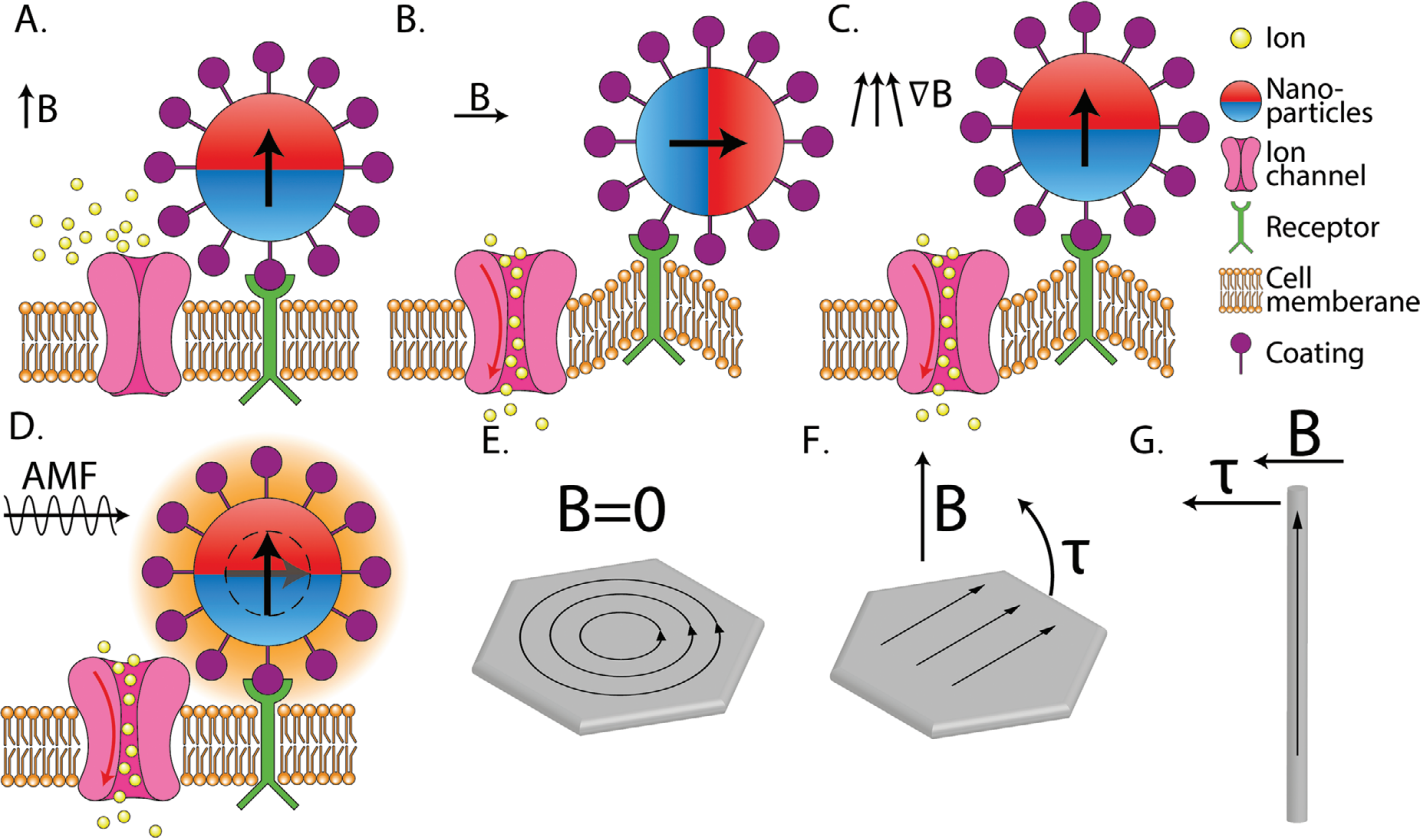
A) The scheme of nanoparticle bound to the cell membrane protein. B) Magnetomechanical neuromodulation mediated by torque from the MNP bound to the protein which responds to the uniform magnetic field, C) MNP in gradient field pulls on bound protein toward the field source and deforms the membrane in magnetomechanical neuromodulation. D) The magnetothermal neuromodulation activates the thermosensitive ion channel through heat dissipation under rapidly alternating magnetic fields. E) Magnetic nanodisc with vortex magnetization in ground state. F) In‐plane magnetization under an external magnetic field thus exerting torque. G) Nano‐bar torque under an external magnetic field.

Before applying a magnetic field, implanted particles can adhere to the cell surface via electrostatic interactions (unspecific site binding)^[^ ^149^, ^150^ ^]^ or anchor to membrane proteins through functional coatings targeting their external domains (specific site binding),^[^ ^151^ ^]^ as shown in **Figure** **5**A. Once the magnetic field is applied, neuromodulation can occur through two mechanisms: magnetomechanical or magnetothermal modulation.

Magnetomechanical neuromodulation utilizes a magnetic field to control the generation of a mechanical force applied to mechanosensory cells. The mechanical force originates from the movement of MNPs in a magnetic field. A magnetic field will be switched on and off or adjusted in terms of direction or magnitude, to force a torque alignment of the nanoparticle toward the external field. Then, the movement of nanoparticles can induce a deformation of the membrane or the mechanosensitive ion channel, which triggers the action potential of the cell. There are mainly two types of mechanical movement in this approach: one involves linear displacement or dragging (as illustrated in the transition from Figure 5A–C), while the other involves rotational or twisting motion (as depicted in the transition from Figure 5A–B).^[^ ^52^, ^152^ ^]^

Magnetothermal neuromodulation utilizes the heat generated by MNPs in response to an Alternating Magnetic Field (AMF) to stimulate cells. When a magnetic nanoparticle is placed in an AMF, its magnetic moment undergoes rapid changes to align with the kHz‐switching magnetic field and generates heat (Figure 5D). There are several physical principles of heat generation. In ferro/ferrimagnetic nanoparticles, the hysteresis loss, caused by shifting of the spin domain walls during the nonlinear realignment process, dissipates heat.^[^ ^153^ ^]^ For superparamagnetic nanoparticles sufficiently small, they will undergo relaxation losses due to their single domain spins, thus producing heat.^[^ ^154^ ^]^ This technique is employed to induce localized heating and is commonly referred to as magnetic hyperthermia, often applied for selective cell destruction, particularly in cancer treatment.^[^ ^155^ ^]^ Optimization of the field parameters and controllable synthesis of MNPs allows a temporary local temperature rise up to 44°C,^[^ ^156^, ^157^, ^158^ ^]^ where the heat applied is tuned to be sufficient to activate thermosensitive ion channels, but without perturbing the membrane and neuron integrity. This approach has been shown to be safe and effective in longitudinal in vivo neuromodulation studies and other clinical MNPs applications such as cancer hyperthermia,^[^ ^159^, ^160^ ^]^ but the clinical translation in the neuromodulation area awaits further systematic safety studies. This approach has already demonstrated usefulness in wireless DBS and applicability in behavioral and therapeutic control of neurological diseases.^[^ ^156^, ^157^ ^]^ Advances with the magnetothermal approach include a demonstration of sub‐second neuromodulation of temporal scale comparable to optogenetics,^[^ ^61^ ^]^ and extend toward transgene‐free approaches to control activity in the deep brain.^[^ ^62^ ^]^

#### 3.3.2 Magnetic Nanoparticles

Nanoparticles serve as the central components of stimulation, and their performance in magnetic fields is primarily influenced by several factors: the choice of materials, size, shape, and crystalline anisotropy.

##### Material

A variety of magnetic materials are employed in the synthesis of nanoparticles, with many being based on iron (Fe) containing compounds. One common approach is to use transition metals or their alloys, such as CoFe.^[^ ^161^, ^162^ ^]^ However, these materials are susceptible to oxidation, which is why iron oxide, particularly magnetite (Fe_3_O_4_) and maghemite (γ‐Fe_2_O_3_), are often favored. Among these, Fe_3_O_4_ is the most widely used choice for MNPs due to its stability, nontoxicity, cost‐effectiveness, and favorable magnetic and biological properties.^[^ ^163^, ^164^, ^165^, ^166^ ^]^

In addition to pure iron oxide, MNPs can be engineered by incorporating divalent transition metal cations, such as Zn^2+^ and Mn^2+.^ This modification allows for the adjustment of the saturation magnetization value of the iron oxide core. For example, nanoparticles like Zn_0.2_Fe_2.8_O_4_,^[^ ^167^, ^168^ ^]^ and (Zn_0.4_Mn_0.6_)Fe_2.8_O_4_^[^ ^169^ ^]^ have been reported to exhibit improved saturation magnetization values compared to iron oxide nanoparticles.

A core‐shell structure is frequently employed in the design of magnetic nanoparticles. In this design, a core material is encapsulated by a shell that imparts additional functions. This brings an extremely diverse combination of various organic, and inorganic materials and is widely applied in bioimaging, biosensors, drug delivery, etc.^[^ ^170^ ^]^ As for MNPs, an example application is the Fe@Fe_3_O_4_ structure. This involves a core of Fe enclosed by a shell of Fe_3_O_4_. This shell can protect the high specific absorption rate of the Fe core from oxidation.^[^ ^171^ ^]^

##### Shape

The shape of nanoparticles plays a crucial role in serval physiological interactions such as toxicity and cellular uptake, but importantly in magnetic neuromodulation, it influences the forces acting on MNPs in a magnetomechanical approach. The particles in general terms can be divided into isotropic and anisotropic groups. The isotropic nanoparticles are faceted with the appearance of a sphere‐like geometry, due to their cubic crystal lattice, and typically exhibit isotropic magnetic behavior, meaning their magnetic properties are uniform in all directions.^[^ ^172^, ^173^ ^]^ This characteristic simplifies their response to external magnetic fields, allowing for predictable and efficient heating profiles during hyperthermia treatment when exposed to gradient magnetic fields. These nanoparticles experience forces proportional to the field gradient, leading to migration toward regions of higher field strength. The moment produced will decrease with the size of the particle reducing and with the distance from the field source increasing.

Anisotropic nanoparticles such as discs, bars, rings, clusters, etc. exhibit specific magnetic properties and can generate magnetic field forces not averagely distributed across the particle, which enables physical movement due to a change of magnetic field direction.^[^ ^174^, ^175^, ^176^ ^]^ Among them, nanodiscs and nanobars are two representative designs. Nanodiscs are typically hexagonal or square‐shaped with diameters ranging from tens to hundreds of nanometers.^[^ ^58^, ^174^, ^175^, ^176^, ^177^, ^178^ ^]^ Their thickness‐to‐diameter ratio gives them an almost two‐dimensional (2D) appearance, which impacts their magnetic properties. These nanodiscs can exhibit vortex spin alignment in their ground state. Their vortex magnetization grants them near zero net magnetization (Figure 5E), allowing for improved colloidal stability by negligent inter‐particle forces, and permits for rotational forces, “torque”. Under slow (1–20 Hz) magnetic fields during the transition to “in‐plane” to align with the magnetic field direction (Figure 5F).^[^ ^58^ ^]^ Nanobars have stick shapes with lengths ranging from 100 to 1000 nm.^[^ ^179^ ^]^ These structures typically feature magnetic poles at both ends, which facilitates exceptional control of their movement under the influence of the torque of an external magnetic field using the lever principle with minimal forces (Figure 5G).^[^ ^180^ ^]^

#### 3.3.3 Stimulus Target

Thermo‐ and mechanosensitive channels serve as promising targets for manipulating neural activity. Thermosensitive channels respond to temperature changes, while mechanosensitive channels react to mechanical forces, by opening the gate and allowing for selective passage of ions thus modulating neuronal depolarization.

The activation of thermosensitive channels is related to certain temperature thresholds or temperature changes.^[^ ^181^ ^]^ Transient Receptor Potential (TRP) superfamily contains many thermosensitive channels. TRPV1 is a common nonselective cation channel used in magnetothermal stimulation. This channel responds to a temperature above the threshold of around 41°C and is expressed in mammals, mostly in the peripheral nervous system.^[^ ^181^, ^182^, ^183^ ^]^ Another common TRP member, TRP member of vanilloid family 4, TRPV4 is also a thermosensitive channel responding to a warm temperature located above 30°C, however, due to its polymodality, it is also actively responding to mechanical stimuli.^[^ ^184^, ^185^, ^186^ ^]^ Similar multimodal channels that respond to several stimuli are TREK 1, TREK2, and TRAAK, which can respond to temperatures above physiological, but with little mechanical sensitivity.^[^ ^187^ ^]^ While the majority of magnetothermal neuromodulation approaches so far relied on targeting the TRPV1 channel demonstrating kinetics on the temporal scale of seconds, more recent studies demonstrate sub‐second control of neuronal activation and even silencing when MNPs are targeted to ion channels activated by sudden change in the temperature such as transient receptor potential ankyrin member 1 (TRPA1) and potassium channel TREK1. Since the latter are sensitive to temperature gradient instead of a temperature threshold, their response to temperature stimuli is faster compared to TRPV1,^[^ ^187^, ^188^, ^189^ ^]^ they demonstrate targets for neuromodulation technology with high spatial and temporal resolution.^[^ ^61^, ^62^ ^]^

The mechanosensitive ion channels can transform mechanical signals into electrical or chemical signals.^[^ ^181^ ^]^ PIEZO1 and PIEZO2 were described as the first mechano‐specific Ca^2+^ permeable mammalian receptors in 2010, with important roles in touch perception, proprioception, and noxious mechanical stimuli.^[^ ^54^, ^190^, ^191^, ^192^ ^]^ They have a special three‐bladed structure, and they deform with the curvature of the membrane, which leads to channel opening, placing them among favorable targets for mechanical stimulation.^[^ ^192^, ^193^, ^194^, ^195^ ^]^ TRPV4 is expressed in various tissues and therefore is a common target for magnetomechanical modulation.^[^ ^184^, ^190^, ^196^, ^197^, ^198^ ^]^ As mentioned before, TRPV4 is however also sensitive to other stimuli, including thermal and chemical signals which may cause plasticity of its mechanical response in vivo.^[^ ^184^ ^]^ TREK‐1 and TRAAK can also respond to mechanical stimuli, but their response is stronger to other stimuli.^[^ ^53^, ^152^, ^184^, ^190^, ^196^, ^197^, ^198^, ^199^, ^200^, ^201^ ^]^ Additionally, there are anionic channels such as cystic fibrosis transmembrane conductance regulator CFTR, which has the potential for mechanical neuronal inhibition.^[^ ^202^, ^203^ ^]^

#### 3.3.4 Coil

There are few special designs of the coil for nanoinvasive neuromodulation technologies. The existing ideas include minisolenoids,^[^ ^49^, ^50^, ^204^, ^205^ ^]^ large solenoids,^[^ ^206^, ^207^ ^]^ and Helmholtz coil(s).^[^ ^208^ ^]^ Some research also generates changing magnetic fields by rotating magnets.^[^ ^209^ ^]^ Most of these designs put tissue or animals under or surrounded by the coil, so the experiments are constrained by the size of the coils. Besides, to account for the field attenuation due to distance, a large current is required to increase the electric field in the region of interest. This also brings unneglectable heat generation, some even need water cooling,^[^ ^157^ ^]^ which is not energy efficient. Therefore, the design of the coil has not received enough attention in this method. Applying microelectronics to nanoinvasive magnetic neuromodulation will be an effective improvement to current research. Millimeter‐scale minicoil array can break the space limitation of experiments by making stimulation devices wearable and moveable and increase energy efficiency by reducing the energy loss in large coils from field attenuation, which are crucial for stable chronic further nanoinvasive magnetic neuromodulation.^[^ ^210^ ^]^

As a summary, **Table** **2** collects literature on nano‐invasive magnetic neuromudulation with listing detailed factors analyized in previous sections over time.

**Table 2:** Publications over time on neuromodulation using MNPs.

Year	Type of Stimulus	Material	Particle size	Shape	Channel	External magnetic source	Refs.
2010	Thermal	MnFe_2_O_4_	6 nm	Nanodisc	TRPV‐1	Solenoid (25 turns, *d* = 7 mm)	[49]
2014	Mechanical	Zn_0.4_Fe_2.6_O_4_	<50 nm	Cube	–	Solenoid (with core, *d* = 1 mm)	[50]
2015	Thermal	Fe_3_O_4_	22 nm	Spherical	TRPV‐1	Solenoid (with C‐shape core, *d* = 4 cm)	[20]
2017	Thermal	Co‐ferrite core at Mn‐ferrite shell	15.65 nm	Core–shell	TRPV‐1	Solenoid	[157]
2020	Mechanical	Fe_3_O_4_	98−226 nm	Nanodisc	TRPV‐4	Solenoid (with core, *d* = 20 cm)	[58]
2021	Thermal	Iron oxide	<25 nm	–	TRPV‐1	Solenoid	[60]
2021	Mechanical	Iron oxide	25 nm	500 nm spherical cluster with nano‐octahedral	PIEZO‐1	Magnets placed on a circular path with *d* = 20 cm	[209]
2021	Mechanical	Fe_3_O_4_	100 nm	–	TRPV‐4	Magnets	[211]
2021	Mechanical	collagen coated Fe_3_O_4_	100 nm‐10 µm	Cluster	–	Solenoid (with core, 1035turns d = 20 mm)	[204]
2022	Thermal	Iron oxide	19 nm	–	TRPV‐1	Solenoid (with core)	[61]
2022	Mechanical	Fe_3_O_4_	212.4 nm, 280.0 nm	Nanodisc	TRPC family	Solenoid (2000 turns, d = 20 mm)	[205]
2023	Thermal	g‐Fe_2_O_3_ core at dextran shell	25 nm	Core–shell	TRPV‐1	Solenoid (17 turns, d = 5 cm)	[212]
2024	Thermal	CoFe_2_O_4_ at MnFe_3_O_4_	14 nm	Core–shell	TREK1	Solenoid	[62]

#### 3.3.5 Synthesis of Magnetite Nanoparticles

Magnetite (Fe_3_O_4_) nanoparticles as mentioned in previous Sections 3.3.2, are the most common material used as central antennae in magnetothermal and magnetomechanical stimulation of neurons.^[^ ^163^, ^164^, ^165^ ^]^ Besides Mn, and Zn‐dopped nanomaterials, magnetite is widely accepted due to its stability against oxidation and its biocompatibility. Therefore, it is worth reviewing the state of the art, and how chemistry has enabled over decades of research, the stabilization of one of the most relevant magnetic nanomaterials.^[^ ^213^, ^214^ ^]^

Magnetite nanoparticles include two general categories which attribute them with specific properties, particularly in heating. Although the synthesis approaches are the same for both, the resulting size leads to significant differences in the magnetic properties. Single‐domain nanoparticles possess a single magnetic domain, wherein all magnetic moments align in one direction. These nanoparticles exhibit high magnetic anisotropy and are efficient at converting magnetic energy into heat through processes like Neel relaxation.^[^ ^215^ ^]^ In contrast, multidomain nanoparticles contain multiple magnetic domains with varying orientations, resulting in lower magnetic anisotropy. Despite their lower anisotropy, multidomain nanoparticles can still generate heat through domain wall movements and Brownian relaxation processes.^[^ ^154^ ^]^

Superparamagnetic iron oxide nanoparticles (SPIONS) are a well‐studied single‐domain material and can be synthesized based on several methods. Most of the methods generate magnetite crystals with a size range between 1 to 100 nm.^[^ ^216^ ^]^ Superparamagnetic nanoparticles have zero net magnetization since each particle corresponds to an individual magnetic domain and no neighbor influence of magnetic dipoles is exhibited. This behavior can be observed as rapid re‐orientation and magnetic saturation in response to an applied magnetic field. The coercivity and remaining magnetization perfectly correlate to the moment when the magnetic field is removed. This rapid transition between a magnetized and demagnetized state is highly desirable in heat‐release applications, like hyperthermia treatment or magnetothermal neuromodulation.

SPIONs can be produced via chemical routes, yielding narrow size, spherical, and colloidally stable suspensions e.g. co‐precipitation, thermal decomposition, microemulsions hydrothermal synthesis, etc. There are top‐down approaches toward the preparation of magnetic materials of controlled shapes and sizes, however, these are usually not below 500 nm and result in lower saturation magnetization^[^ ^217^ ^]^ compared to wet chemistry approaches. The most popular synthesis route, due to low cost and time consumption, corresponds to co‐precipitation in the presence of reducing agents. The oxidation state of Fe (II) in the crystal lattice of magnetite is relevant to achieving high magnetic saturation states (near 100 emu g‐1 for 20 nm diameter particles), thus, the use of reducing agents (NaOH, ammonia, TMAOH, etc.) under anoxic conditions are required for high crystallinity.^[^ ^218^, ^219^ ^]^ However, the nature of coprecipitation steps exhibits poor control over nucleation, leading to suspensions with polydisperse size distributions, as well as low colloidal stability in aqueous or nonpolar solvents. To overcome these problems, further coating steps with ligands such as citric acid or oleic acid enables a better dispersion in water and organic solvents. Especially the use of oleic acid and sodium oleate has allowed the synthesis of Fe‐oleate precursors to control nucleation steps and avoid abrupt phase transitions. Such methodologies are referred to as thermal decomposition and typically generate spherical, narrow‐size suspensions of magnetite nanoparticles with controllable diameters of 5–30 nm. Thermal decomposition is characterized by the degradation of iron precursors in the presence of oleic acid, which at a high temperature and reducing conditions can smoothly control the nucleation times by forming Fe‐oleate micelles where nanocrystals can grow. The size of the micelles controls tightly the maximum diameter of the particles leading to near monodisperse suspensions.

Magnetite of anisotropic shapes can deliver mechanical stimuli to the neuronal membranes due to ground vortex magnetization and realignment under the uniform magnetic field as mentioned previously. However, anisotropic nanoparticles of the stable magnetite pose a challenge due to their inverse spinel lattice with cubic symmetry, which inherently favors isotropic growth. The approach toward the synthesis of anisotropic magnetite nanoparticles with vortex is through the preparation of the hematite templates. Hematite (Fe_2_O_3_) is the nonmagnetic form of iron oxide with a hexagonal crystal lattice and allows anisotropic growth, as in the example of the hydrothermal synthesis of hematite hexagonal disk.^[^ ^220^ ^]^ Hydrothermal synthesis relies on solubility changes of chemical species under high temperatures and pressure, leading to complex nucleation paths unable to be achieved under normal solvothermal systems.^[^ ^220^ ^]^ Under high pressure and temperature, layers of solvents in the presence of counter anions (acetate) drive the crystal growth of hematite, shaping hexagonal morphologies with diameters up to 230 nm. The content of water in the reaction controls the growth of the crystal due to strict competition on binding sites, which dictate the axis growth of each individual particle.^[^ ^220^ ^]^ Furthermore, the in‐plane growth of the disks can be controlled by adding different counter ions as sulfate or phosphate, favoring stable rings and tube structures.^[^ ^221^ ^]^ The mechanism of crystal growth in such as complex system has not been studied in‐depth and remains a challenge to be tackled by the community. Although complex anisotropic hematite nanoparticles can be achieved via hydrothermal synthesis, the magnetic properties are only exhibited after a reduction step, where H_2_ is used to transfer electrons into a crystal structure rich in Fe (III) in the presence of a co‐reducing agent, such as trioctylamine. Finally, with an increase in reaction temperature to reflux (up to 370°C), the magnetite crystal phase is obtained from original hematite templates. The anisotropic nanoparticles are usually stabilized by incorporating oleic acid into the reduction reaction, which allows their dispersion in organic solvents and allows transfer into water solution by ligand replacement or surface coating steps.^[^ ^220^ ^]^

### 3.4 Overview of Magnetic Neuromodulation Technologies

The previous sections have provided a comprehensive overview of the three magnetic neuromodulation methods tabulated in **Table** 3. In principle, noninvasive and invasive methods are based on induced electric fields, while nanoinvasive methods involve the movement or heating of nanoparticles. The spatial resolution and performance of DBS of invasive and nanoinvasive methods are significantly better than that of noninvasive methods. However, invasive and nanoinvasive methods require invasiveness. Although the nanoinvasive method is less invasive, it is also the only method that requires genetic engineering.

**Table 3:** Comparison between three methods.

Property	Noninvasive	Invasive	Nanoinvasive
Principle	Stimulate by magnetic field induced electric field	Stimulate by magnetic field induced electric field	Stimulate by movement or heating of nanoparticles
Spatial resolution	Tens of cm	Hundreds of µm	Specific neuron(s)
Invasiveness	No	High	Low
Genetic engineering	No	No	Yes*
Deep brain Stimulation accuracy	Low	High	High
Coil size	Centimeter	Sub‐millimeter	Millimeter to centimeter

* *Transgene‐free methods for PNS stimulation are available. Transgene‐free methods for CNS reported*.^[^ ^62^ ^]^

## 4 Microfabrication Technologies for Magnetic Neuromodulation

Building on the previous sections that highlighted the development of next‐generation high‐selectivity magnetic stimulation and the crucial role of mini/microcoils, this section explores state‐of‐the‐art microfabrication techniques essential for achieving coil miniaturization. The fabrication methods discussed include through‐silicon via (TSV), surface micromachining, lithography, direct writing, and 3D printing. Each technique will be examined in terms of its fundamental principles, materials, and applications in microcoil fabrication. While some of these technologies have yet to be applied in neurostimulation (particularly in nanoinvasive approaches), they hold significant potential for future applications, especially in specific modulation scenarios.

### 4.1 The Fabrication Process Involving Through‐Silicon Vias

TSV technology uses high aspect ratio etching and deposition techniques to create openings ranging from sub‐micrometers to several hundred micrometers.^[^ ^222^ ^]^ This technology efficiently accesses and modulates high‐density coils fabricated on the same substrate, a process commonly used in CMOS foundries. TSV involves creating vias in a silicon or glass substrate. For conductive substrates, insulation layers are added to electrically isolate TSVs and prevent insertion losses.^[^ ^223^ ^]^ The vias are then filled with conductive materials such as doped polysilicon, low‐resistivity monocrystalline silicon, tungsten, or copper.^[^ ^224^, ^225^, ^226^, ^227^ ^]^

**Figure 6:**
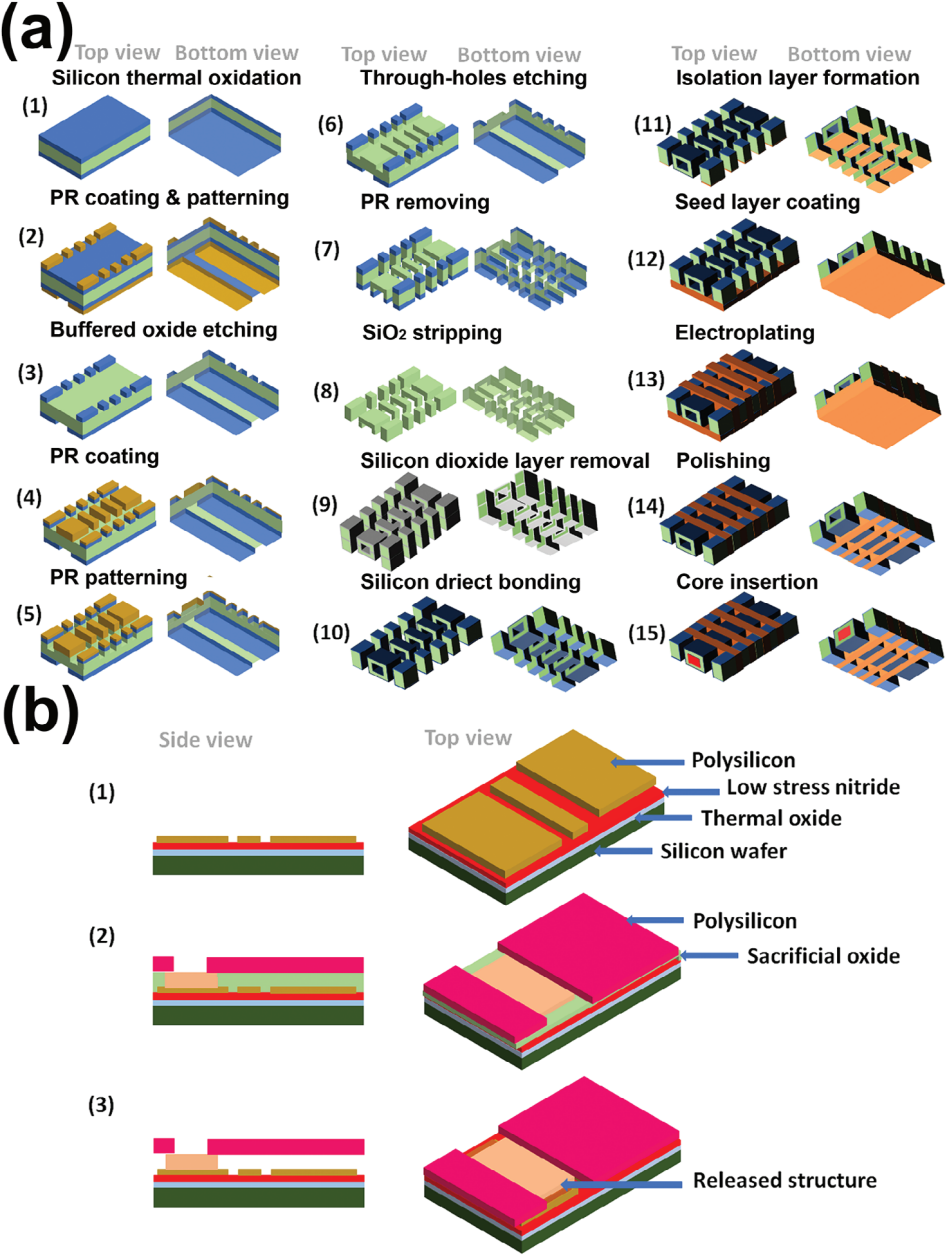
a) Schematic of TSV process for microcoils (1 mm high, 100 µm side, and 100 µm gap) fabrication; 1) silicon dioxide layers formation using a thermal oxidation method, 2) photoresist (PR) coating and patterning, 3) a buffered oxide etching, 4) another PR coating and patterning, 6) through‐holes etching, 7) PR removing, 8) a mask layer coating, through‐hole etching, 9) removing all of the silicon dioxide layer, 10) process of direct bonding of the silicon to form spaces for iron cores in the case of solenoid coils with the core, 11) isolation layer formation using silicon dioxide layers between the substrate and coil, 12) a seed layer coating, 13) copper electroplating, 14) through silicon electroplating of copper and a polishing, 15) iron core insertion. Reproduced under terms of the CC‐BY license.^[^ ^228^ ^]^ Copyright 2019, IEEE b) schematic of the manufacturing process of a surface micromachined constructs (200 µm × 200 µm × 1 µm); 1) the ground plane and insulating materials deposition, 2) deposition of polysilicon and sacrificial oxide, 3) released structure. Reproduced under terms of the CC‐BY license.^[^ ^247^ ^]^ Copyright 2003, Elsevier.

**Figure** **6**a presents a schematic (not to scale) of a two‐stage TSV process for fabricating solenoid inductors with integrated iron cores. In the first stage, inductor and core geometries are defined within a double‐side‐polished silicon substrate. This process includes thermal oxidation, photolithography, and etching to form through‐holes, trenches, and the core cavity. The core is then sealed within the substrate using silicon direct bonding. In the second stage, copper coils are formed via electroplating. A thick oxide layer is grown for insulation, followed by seed layer deposition and “bottom‐up” filling of the trenches and vias. Excess copper is removed by grinding, and the inductors are separated by dicing. Finally, an iron core is inserted into each inductor to complete fabrication.^[^ ^228^ ^]^ The fabricated inductors have a height of 1 mm, square copper coils with 100 µm sides, and 100 µm gaps between the coils.^[^ ^228^ ^]^ TSVs are classified as via‐last, via‐middle, or via‐first based on their process sequence in the overall manufacturing process.^[^ ^222^ ^]^

TSV technology is primarily applied to producing 3D toroidal and 2D spiral coils.^[^ ^229^, ^230^, ^231^, ^232^, ^233^ ^]^ Toroidal coils consist of two conductor layers and a central magnetic layer, while spiral coils consist of two magnetic layers encasing the winding. Both types of coils involve multiple layers and require a higher number of TSVs, increasing the contact interfaces and potentially reducing structural robustness.^[^ ^234^ ^]^ The difference between silicon‐based toroidal and spiral coils is that the core thickness is limited by the thickness of the wafer, and this is circumvented in the case of spiral coils. Considering strict microfabrication restrictions, including low via size and substrate thickness, a spiral design at the millimeter scale usually has a reduced DC resistance or increased inductance density than a toroidal design.^[^ ^235^ ^]^ For example, a TSV‐fabricated spiral coil with a thickness of 200 µm can carry up to 5A of DC current while maintaining a DC resistance of 23 mΩ.^[^ ^230^ ^]^ Additionally, 3D solenoid coils fabricated in silicon using TSVs, such as a 15‐turn solenoid with an iron core, have demonstrated high inductance densities of 354.3 nH mm^−2^.^[^ ^228^ ^]^

A notable advancement in neuromodulation using TSV technology is the development of an 80 µm × 40 µm microfabricated solenoid coil, integrated with a magnetic core to enhance magnetic flux over air‐core designs. This coil was engineered for neuronal tissue activation and evaluated using *ex vivo* calcium fluorescence imaging (GCaMP_6s_).^[^ ^119^ ^]^ Calcium dynamics were monitored in acute brain slices (300 µm thick) from 15‐ to 30‐day‐old Thy1‐GCaMP_6s_ transgenic mice. The microsolenoid was positioned at a depth of ≈170 µm within the brain slice, enabling highly localized neuronal activation, and demonstrating the coil's capacity for high‐resolution stimulation.^[^ ^119^ ^]^

Another study using these microcoils investigated the modulation of cortical pyramidal neuron (PN) activity.^[^ ^115^, ^236^ ^]^ Electrophysiological recordings were performed on acute coronal brain slices (300‐400 µm thick) from the prefrontal cortex (PFC) and primary motor cortex (M1) of C_57_BL/6J mice aged 17 to 30 days. Researchers examined how microcoil orientation affected the activation of layer 5 PNs in the PFC and M1.^[^ ^115^ ^]^ To assess neuronal responses, cell‐attached patch clamp recordings were conducted on a total of 103 cells across 43 brain slices. For each experiment, position the microcoil at a constant height of 100 µm above the slice and ensure an initial distance of 100 µm between the coil's proximal edge and the soma of the targeted PN. The maximum voltage applied to the coil without overheating was ≈40 V. At this input level, the electrical artifacts produced by both pulsatile and sinusoidal stimulation closely mirrored the time derivative of the input waveform. The resulting peak magnetic field strength, peak electric field strength, and peak field gradient were measured to be 0.3 T, 10.7 V m, and 23 kV m^−^^2^, respectively. Repeated stimulation effectively triggered activity, and layer 5 PNs showed robust activation. The asymmetric magnetic fields generated by the coils created a confined activation pattern, preventing the excitation of orthogonally oriented axon fibers as mentioned in Section 3.2.1.^[^ ^115^ ^]^

### 4.2 Surface Micromachining

Surface micromachining involves fabricating structures layer‐by‐layer through the deposition of thin films on substrates as functional compounds.^[^ ^237^ ^]^ This technique is commonly used in producing microcoils with high precision in a clean‐room‐compatible microscale‐to‐nanoscale range. It comprises of deposition of thin films and sacrificial layers, photolithography, and etching steps.^[^ ^238^, ^239^, ^240^ ^]^ Silicon is a common choice as a substrate due to its compatibility with microfabrication processes, but other materials such as glass also have been utilized as well.^[^ ^241^, ^242^ ^]^ The deposition of thin films onto the substrate is performed through techniques like chemical or physical vapor deposition with selected materials.^[^ ^243^ ^]^ Sacrificial layers are temporarily deposited and subsequently removed to release the final microstructure, which is deposited between the coil windings.^[^ ^244^ ^]^ Following thin film deposition, a photoresist is patterned to define the intended structure after which the sacrificial layer is etched away via wet or dry etching.^[^ ^238^, ^239^, ^240^, ^243^, ^245^, ^246^ ^]^

Figure 6b (not to scale) outlines the surface micromachining process on a silicon substrate. The process starts with thermal oxidation (0.6 µm), silicon nitride deposition (0.8 µm), and patterning of phosphorous‐doped polysilicon. A 2 µm sacrificial oxide layer is then deposited, followed by structural polysilicon deposition (0.8 µm), patterning, and annealing. Finally, the sacrificial oxide is etched with hydrofluoric acid, releasing the microstructures from the substrate. The final resonator has dimensions of ≈200 µm × 200 µm × 1 µm.^[^ ^247^ ^]^

Since the advent of 2D surface micromachining (2D‐SM), several studies have been conducted to fabricate various microscale solenoid, spiral, and 2D planar coils.^[^ ^239^, ^240^, ^248^ ^]^ 2D‐SM has been particularly used in the manufacturing of on‐substrate low‐aspect‐ratio planar coils relying on molding or sacrificial layers or a mix of both approaches.^[^ ^240^, ^249^, ^250^ ^]^ Nonetheless, there are challenges associated with 2D coils, such as the low quality factor of 2D planar coils, which can be enhanced by air bridges, and thick metal or shielding layers.^[^ ^239^, ^240^, ^251^ ^]^ Besides, as the primary axis of the coil in 2D spiral coils is perpendicular to the substrate, the magnetic flux density is also perpendicular to the substrate. When the substrate has lossy characteristics such as free charges, the magnetic flux might generate eddy currents as depicted in Section 3.1 and as a result, increase the temperature of the substrate and reduce the effective magnetic field due to current confinement to the edges of the coil at higher frequencies.^[^ ^252^, ^253^, ^254^ ^]^ Complete eradication of eddy currents can be achieved by removal of the substrate beneath the coil or by utilization of high resistivity Si substrates.^[^ ^255^ ^]^

3D surface micromachining (3D‐SM) is utilized for the production of high‐performance spiral microcoils.^[^ ^256^ ^]^ The magnetic flux in the aforementioned usually runs parallel to the surface of the substrate. This leads to lesser flux within lossy substrates and a reduction in eddy currents, leading to an improvement in the quality factor.^[^ ^255^, ^257^, ^258^ ^]^ To mitigate the parasitic effects of the substrate and improve magnetic induction, constructs with 3D characteristics are preferable.^[^ ^255^, ^257^, ^258^ ^]^ 3D coils with soft ferromagnetic cores are better equipped to confine the magnetic field more effectively, minimizing energy dissipation.^[^ ^256^ ^]^ This method allows the creation of intricate 3D coil geometries, overcoming the limitations of planar coils by improving magnetic field confinement and minimizing energy dissipation within the surrounding tissue. These attributes are particularly crucial for achieving precise and focused neurostimulation and minimizing unwanted side effects. Recent studies have showcased the capabilities of 3D‐SM‐fabricated microcoils in eliciting selective neuronal activation, both in vitro and in vivo.^[^ ^19^, ^41^, ^146^, ^259^ ^]^

For example, a microcoil design featuring a copper trace (10 µm wide × 2 µm thick) on a silicon substrate (cross‐sectional area 50 µm × 100 µm, length 2000 µm) was developed to investigate neuronal activation and behavioral responses.^[^ ^41^ ^]^ In vitro experiments on mouse brain slices confirmed reliable, spatially confined stimulation (<60 µm). The ability to manipulate coil orientation further enhanced neuronal targeting selectivity due to the asymmetric field generated, reducing the co‐activation of axons. In vivo implantation in the mouse whisker motor cortex was safe and elicited predictable behavioral responses, with whisker movement direction dependent on stimulation frequency, mirroring results from electrical stimulation.^[^ ^41^ ^]^ This study has shown that magnetic stimulation from a microcoil can selectively activate PNs while avoiding passing axons,^[^ ^41^ ^]^ prompting further exploration of how microcoil design influences neuronal activation selectivity.

Beyond simple geometries like rectangular (Figure 4D1), the exploration of more complex coil designs, such as V‐ and W‐shaped coils (Figure 4D2,3), has revealed their superior selectivity in activating specific neuronal populations.^[^ ^19^ ^]^ Electrophysiological experiments, including patch‐clamp recordings and calcium imaging, confirmed the reliable activation of layer 5 PNs by both V‐ and W‐shaped coils, with V‐coils exhibiting greater efficacy and W‐coils demonstrating enhanced selectivity. The influence of coil geometry on activation thresholds has also been observed, with double‐loop coils demonstrating lower thresholds compared to single‐loop coils, and calcium imaging demonstrated superior spatial confinement of activation compared to traditional electrodes.^[^ ^19^ ^]^ Furthermore, in vivo experiments using implantable microcoils of varying geometries, such as V‐shaped and semicircular, were conducted to achieve localized brain stimulation on a micrometer scale.^[^ ^146^ ^]^ In vivo experiments in 6‐ to 10‐week‐old male C_57_BL/6J mice confirmed neural activation, with the strongest response elicited by a 70 µm concentric coil. Importantly, this study revealed frequency‐dependent effects of µMS, with lower frequencies (≤0.1 kHz) exciting neural tissue and higher frequencies (>0.1 kHz) inhibiting action potential propagation.^[^ ^146^ ^]^ These findings align with established principles of electrical stimulation and further support the potential of microcoils for achieving controlled and targeted neuromodulation. The ongoing exploration of novel coil designs, including those inspired by the “figure‐of‐8” coil used in TMS, aims to further optimize the spatial control and efficacy of magnetic neurostimulation.^[^ ^260^ ^]^ By strategically combining multiple microcoils and adjusting their relative distances, it could be possible to optimize both the magnetic/electric field strength and its spatial derivative. This approach, building upon the principles of “figure‐of‐8” coils, presents a promising avenue for developing microscale devices with tailored stimulation profiles.^[^ ^260^, ^261^, ^262^ ^]^

### 4.3 3D Micro/Nanofabrication Methods

The drive toward creating 3D structures through additive manufacturing processes has been instigated by the need for high‐resolution, ecofriendly fabrication techniques that do not heavily rely on hazardous materials and chemicals.^[^ ^263^ ^]^ Within these methodologies, a variety of techniques such as lithography, direct writing, and 3D printing have been employed to manufacture a spectrum of 3D nanostructures,^[^ ^264^, ^265^ ^]^ which are discussed in the following sections.

#### 4.3.1 Lithography

Lithography is a methodology employed for creating structures at the nanoscale or microscale. By exposing the photoresist under a source of light going through a mask with the desired pattern.^[^ ^266^ ^]^ The pattern size is limited to the exposure wavelength and sub‐micrometer features are developed via electron‐beam lithography. The substrate undergoes a coating process with single or multiple layers of functional compounds serving as a hard mask, dielectric, or conducting layer.^[^ ^267^ ^]^ Resists are commonly categorized as negative or positive and amplified or nonamplified resists including polymethylmethacrylate, SU‐8, and novolac‐epoxy resin.^[^ ^268^, ^269^ ^]^ Besides, there is the use of composite materials, combining gold and silver salts with photoresists for metal deposition.^[^ ^270^ ^]^ In general, lithography processes form a pattern in the resist, followed by the pattern transfer to the substrate through processes such as lift‐off or etching.^[^ ^246^ ^]^

**Figure 7:**
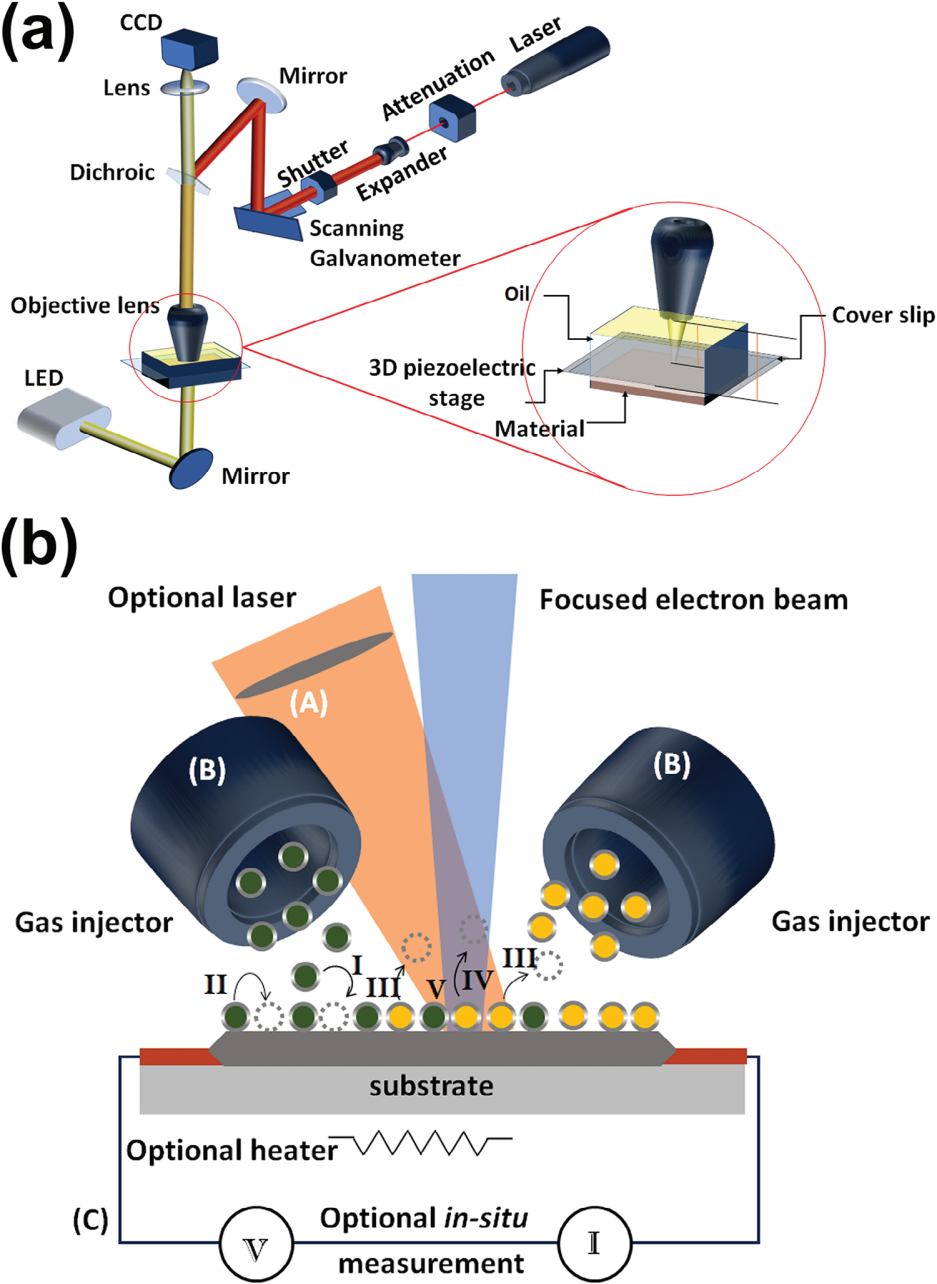
Schematic of a a) two‐photon polymerization schematic system setup. Reproduced under terms of the CC‐BY license.^[^ ^271^ ^]^ Copyright 2017, AIP Publishing; b) scanning electron microscopy adapted for focused electron beam‐induced deposition with some extra equipment; A) utilizing either pulsed or steady‐state infrared lasers to supply heat to improve the desorption of unwanted products of organic dissociations, B) deposited layers characterization in situ unit, C) multichannel gas injection precursor system for multicompounds deposition (organic ligands represented by gray color metal shown either by red or blue color). Numbers 1 to 5 stand for surface physio‐adsorption, surface diffusion, desorption induced by heat and electron, and organic ligands desorption, respectively. Reproduced under terms of the CC‐BY license.^[^ ^284^ ^]^ Copyright 2017, Elsevier B.V.

For precise micropatterning on a substrate surface, various lithography techniques are deemed effective, including photolithography, X‐ray lithography, and two‐photon polymerization (TPS).^[^ ^246^, ^267^, ^271^ ^]^ TPS provides superior process flexibility and resolution, enabling the fabrication of constructs with dimensions smaller than 100 nm.^[^ ^272^ ^]^ Another advantage of employing TPS is its capability to print within the resin, extending beyond surface limitations. However, due to its reliance on a focused laser beam and a spot scanning approach, TPS exhibits a lower processing speed when compared to other lithography methods.^[^ ^273^ ^]^ The other challenge linked with TPS is the occurrence of intralayer surfaces of the stair‐stepping.^[^ ^274^ ^]^ The schematic of the TPS system is shown in **Figure** **7**a. The fabrication of high aspect ratio cylindrical microcoils on an acrylic substrate, featuring a 20 µm pitch and a 10 µm width has been documented through X‐ray lithography.^[^ ^275^ ^]^ However, the drawback of this method lies in the necessity for an expensive and large synchrotron radiation facility to serve as the light source. To benefit from the advantages of the TPS method, the fabrication of 3D microcoils composed of a hybrid material, incorporating both a “metal‐coatable polymer” and a polymer has been reported.^[^ ^276^ ^]^ Selective metallization of the metal‐coatable polymer microstructure was accomplished through the process of electroless silver plating. The resulting multi‐turn 3D microcoil, with a diameter and height of 200 µm, and 60 µm, respectively, was found to be operational at a frequency of 25.4 GHz.^[^ ^276^ ^]^

In magnetic neuromodulation, Lithography techniques have emerged as a powerful tool in the fabrication of mini/microcoils especially and paved the way for the development of highly precise mini/microcoil arrays and probes. The ability to precisely pattern conductive materials on a substrate using lithography processes allows for the creation of intricate coil geometries with well‐defined dimensions, leading to highly localized and controlled magnetic fields.^[^ ^277^ ^]^ This spatial confinement is crucial for minimizing off‐target effects and achieving selective stimulation of specific neuronal populations.^[^ ^278^ ^]^ In vitro investigations have utilized lithography‐based micromagnetic platforms, featuring a 6×6 array of microcoils, to achieve remarkable spatial precision in stimulating neural cells.^[^ ^141^, ^279^ ^]^ These microcoils, capable of generating magnetic flux profiles with a resolution of 100 µm, successfully induced sufficient potentials to excite neural cells even at a distance of 30 µm, demonstrating their efficacy in eliciting neural responses.^[^ ^141^, ^279^ ^]^ Furthermore, photolithography has enabled the fabrication of miniature microcoil probes directly on silicon wafers.^[^ ^259^ ^]^ The manufactured probes had dimensions of 60 µm in thickness, 4 mm in length, and as narrow as 150 µm, with a coil wire width of 15 µm. The device exhibited a DC resistance of 4 Ω and a leakage resistance exceeding 210 GΩ. The recorded potentials evoked cortical potentials closely resembled both the duration and amplitude of light‐induced visual evoked potentials in response to magnetic stimulation,^[^ ^259^ ^]^ suggesting their potential for precise neuromodulation. Moreover, the ability to manipulate coil orientation further enhances neuronal targeting selectivity due to the asymmetric field generated, reducing unwanted activation of nearby axons.^[^ ^280^ ^]^

In addition to spatial precision, lithography‐fabricated minicoils have also been shown to modulate synaptic plasticity, a key mechanism underlying learning and memory.^[^ ^281^, ^282^ ^]^ To this end a study used photolithography to fabricate a planar coil with a line width and spacing of 30 µm, an inner diameter of 2 mm, an outer diameter of 11 mm, and 75 turns. This coil was part of a contact‐mode magnetic stimulation platform, where magnetic stimulation was applied to hippocampal slices through the coil positioned underneath in contact mode. The fabricated coil had an inductance of 26.3 ± 1.5 µH, a capacitance of 1.31 ± 0.2 pF, and a resistance of 31.02 ± 1.7 Ω. Field excitatory postsynaptic potentials were evoked by synaptic input in the Schaffer collaterals pathway and CA_1_/Subiculum and recorded at the CA_1_ stratum radiatum. After 60 V contact‐mode magnetic stimulation, the field excitatory postsynaptic potentials slope showed minor changes from the baseline in the Schaffer collaterals pathway, while it was 5% lower than the baseline in Subiculum. With 70 V, the field excitatory postsynaptic potential slopes declined by 14% and 16% in the Subiculum and Schaffer collaterals pathway, respectively. At 80 V, significant reductions in field excitatory postsynaptic potential slopes were observed, with 37% in Subiculum and 38% in the Schaffer collaterals pathway. The synaptic response increased with the intensity of magnetic stimulation, and a long‐term depression‐like effect was observed in CA_1_ by synaptic input Subiculum and Schaffer collaterals pathway after stimulation of the entire hippocampal slice.^[^ ^283^ ^]^ This finding highlights the potential of mini/microcoils for not only stimulating neuronal activity but also influencing synaptic strength and plasticity, opening new avenues for therapeutic interventions in neurological disorders associated with impaired synaptic function.

#### 4.3.2 Direct Ink Writing

Focused electron beam‐induced deposition (FEBID) is a direct‐writing approach that exploits the direct deposition of structures on the surface of a substrate by scanning a focused electron beam across the desired pattern of the construct in the presence of a precursor gas. Typically performed within electron microscopes, this method facilitates easy in‐situ inspection of the manufactured construct.^[^ ^285^, ^286^ ^]^ In addition to deposition, other effects such as in‐situ heating or etching can be performed using the electron beam. Considering the focus‐ability of electron beams in the range of micrometers to sub‐angstrom, this process is appropriate for applications in both the micro‐ and nanometer scales.^[^ ^287^ ^]^

In FEBID, precursors are delivered through a capillary tube to provide a continuous supply of molecules in gaseous form to the surface. A portion of the incoming flux is then chemo‐ or physiosorbed on the surface of the substrate.^[^ ^288^ ^]^ The precursor molecules dissociation can occur due to the interaction of backscattered, secondary, and primary electrons with the adsorbed molecules on the surface.^[^ ^285^, ^289^ ^]^ Typically, the precursors are complexes comprising ligands and a metal core named organometallic precursors, where the selection of the precursor type primarily determines the composition of the deposited compound.^[^ ^284^, ^285^ ^]^ For instance, the most employed precursors for the fabrication of ferromagnetic 3D nanostructures are dicobalt octacarbonyl, iron pentacarbonyl, and diiron nonacarbonyl.^[^ ^290^ ^]^ FEBID can deposit superconducting alloys, metallic materials, metamaterials, and intermetallic compounds, including gold, silver, tungsten, iron, ferro‐cobalt alloys, cobalt, and platinum.^[^ ^284^, ^285^ ^]^ The Schematic diagram of FEBID is shown in Figure 7b.

FEBID has the capability of depositing materials on nonflat substrates and is geometrically intricate, which might be unsuitable or challenging for other deposition methods.^[^ ^286^ ^]^ FEBID has been successfully employed to deposit magnetic nanowires onto the apex of AFM cantilever tips, thereby functionalizing them for magnetic force microscopy.^[^ ^291^, ^292^ ^]^ This demonstrates the potential of FEBID in tailoring nanoscale probes for specialized applications. In FEBID there is a possibility to direct the beam in a way to enables continuous growth of the desired compounds over a specified site to induce additional deposition on the previously grown compound, facilitating the efficient patterning of 3D nanoconstructs,^[^ ^293^ ^]^ where helical nanostructures fabrication with the capability to function as nanocoils has been reported in.^[^ ^294^ ^]^ By enabling the deposition of functional nanostructures with intricate 3D geometries, 3D nanopatterning provides a platform for investigating the divergent properties of materials resulting from distinct deposition processes. This includes elucidating the differences in electrical behavior, microstructure, and composition observed based on employed techniques.^[^ ^295^, ^296^, ^297^, ^298^ ^]^ The applicability of FEBID has been showcased in several fields, including the repair of defects in optical lithography masks, targeted deposition of functional materials, and post‐fabrication modification of circuits, highlighting its potential as a versatile nanofabrication tool.^[^ ^299^, ^300^ ^]^ The fabricated structures demonstrate typical Josephson Junction behavior, exhibiting Shapiro steps in the current–voltage characteristics under 6.4 GHz microwave irradiation and an Ic(B) dependence akin to a Fraunhofer diffraction pattern. The superconducting and normal conducting regions in these structures are created by modulating the electron beam current during deposition.^[^ ^301^ ^]^ Considering the proven capability of FEBID in fabricating intricate 3D magnetic nanostructures, this technology shows great promise for the nanoprinting of 3D nanostructures. Significant advancements and applications are anticipated in the coming years. However, for neurostimulation applications, a restrictive factor is the need for high throughput FEBID with higher functioning speed as current systems operate at a speed of tens of nanometers per second. This especially is observed as a critical factor in tall constructs and long parts where a significant reduction in growth rates has occurred.^[^ ^285^, ^302^, ^303^ ^]^

Although not yet applied to neuromodulation, FEBIDs present a promising avenue for the future fabrication of magnetic neurostimulation coils. FEBIDs offer unique capabilities, such as precise 3D nanopatterning and deposition on nonplanar substrates, which could enable highly customized and complex coil designs. These coils could provide superior spatial resolution and localization compared to traditional methods, leading to more effective and focused neurostimulation therapies.

#### 4.3.3 Microcoils Printing

As already mentioned, the conventional approach for producing microcoils is both costly and time‐consuming. Nevertheless, there are highly rapid, consistent, and cost‐effective methods for manufacturing microcoils through additive manufacturing techniques also known as digital writing and direct printing.^[^ ^304^ ^]^ These processes such as screen printing, inkjet printing, electrohydrodynamic printing, and aerosol jet printing are utilized to create parts with precise control over their composition and architecture for a wide range of applications at both the macro‐ and microscale.^[^ ^305^, ^306^, ^307^, ^308^ ^]^

##### Screen Printing

**Figure 8:**
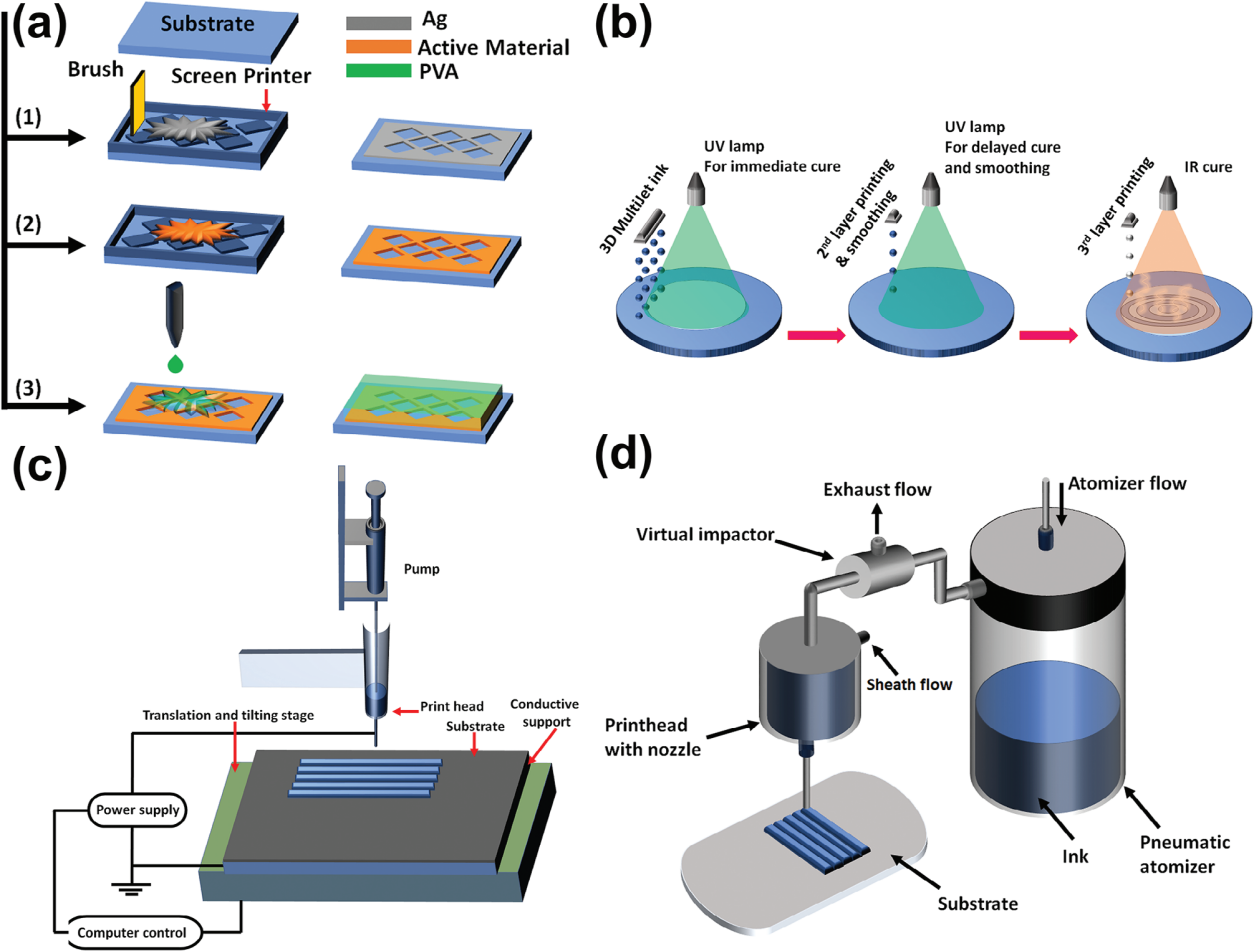
Schematic presentation of a) screen printing Reproduced under terms of the CC‐BY license.^[^ ^310^ ^]^ Copyright 2014, AIP Publishing; b) inkjet printing Reproduced under terms of the CC‐BY license.^[^ ^314^ ^]^ Copyright 2017, Springer Nature c) electrohydrodynamic printing Reproduced under terms of the CC‐BY license.^[^ ^321^ ^]^ Copyright 2007, Springer Nature; and d) aerosol jet printing Reproduced under terms of the CC‐BY license.^[^ ^322^ ^]^ Copyright 2022, Springer Nature.

Screen printing is one of the commonly employed approaches in electronics manufacturing. This technique can print a desired design on a flat surface using a squeegee, ink, and a mesh screen.^[^ ^309^, ^310^ ^]^ The fundamental process forms a stencil on a fine mesh screen and subsequently uses a squeegee to paint, thereby generating an imprint of the design on the underlying substrate. It can be utilized on surfaces of any size and shape, including eyeglasses, inner surfaces, or windshields, distinguishing it from inkjet technologies.^[^ ^309^ ^]^ The fabrication of microcoils via screen printing involves three key steps, as depicted in **Figure** **8**a: electrode printing, active material printing, and electrolyte deposition. Initially, electrodes (e.g., silver) are screen‐printed onto a bare substrate such as polyethylene terephthalate, followed by drying and annealing in a vacuum oven. Subsequently, the active material ink (e.g., MnO_2_/onion‐like carbon) is printed atop the silver electrodes and dried. Lastly, an electrolyte sol such as polyvinyl alcohol /H_3_PO_4_ is coated over the device to encapsulate the channel area and then air‐dried to complete the fabrication process.^[^ ^310^ ^]^ Employing this method, a microinductor with a low profile was created on a substrate of copper‐cladded polyimide. The coils carrying the current were designed from the pre‐existing metallization layer, and using a composite of ferrite‐polymer the magnetic core was printed.^[^ ^305^ ^]^ A study investigated the viability of graphene nanoplatelet‐printed electrodes for cortical direct current stimulation, comparing them to traditional silver/silver chloride pellet electrodes.^[^ ^311^ ^]^ The study found that while silver‐based electrodes pose a risk to living tissue due to redox reactions caused by partial permeability of the printed layers, graphene nanoplatelet‐based electrodes are electrochemically safe for direct neural stimulation.^[^ ^311^ ^]^ As another proof of concept, a screen‐printed masking technique using polyvinyl chloride ink was employed to fabricate a copper‐based microelectrode array patch, with an integrated pH sensor for monitoring blood pH levels.^[^ ^312^ ^]^ Simulations optimized electrical stimulation parameters, focusing on varying DC potentials to generate effective electro‐taxis. The flexible and skin‐conformal patch demonstrated good electrical continuity. When applied to cutaneous wounds in rats, the patch significantly accelerated wound healing, reducing healing time to 9 days compared to 13 days for controls.^[^ ^312^ ^]^ The flexible, screen‐printed electrodes meet the requirements for subdural implantation or wearable applications, offering a promising alternative for future magnetic brain stimulation coil fabrication. While screen printing has not yet been applied to fabricating coils for magnetic neurostimulation, its demonstrated ability to create flexible, biocompatible, and precisely patterned electrodes makes it a strong candidate for microcoil fabrication in neuromodulation.

##### 3D Inkjet Printing

3D inkjet printing is a process that operates at low pressures and temperatures, involving the laying down of either suspension of solid or liquid substances such as dielectric nanoparticles, polymers, and conductive nanoparticles. In this technique, the printing compounds are extruded via a nozzle, while the print head moves back and forth across a surface in a raster pattern, constructing multiple layers one after the other in a sequential layering process, which is shown in Figure 8b. The primary methods of inkjet printing include drop‐on‐demand mode and continuous inkjet mode.^[^ ^302^, ^303^, ^313^, ^314^ ^]^ Drop‐on‐demand printing is recognized for its capability in the fabrication of constructs composed of multi‐materials, due to its technological flexibility in dynamically adjusting to various materials and patterns. This is in contrast to other manufacturing and printing processes, which typically necessitate fixed tooling or masks.^[^ ^315^ ^]^ While this printing method stands out as scalable, noncontact, and less prone to issues like contamination and damage to masks or substrates, the achievable resolution and range of possible shapes in fabrication were restricted.^[^ ^316^ ^]^ For instance, this method has been used to print a flexible microinductive coil composed of copper on a paper substrate.^[^ ^306^ ^]^ In another study, a combination of electroplating and printing methods was used to fabricate a 16‐turn solenoid on microcapillaries, measuring 1.6 mm in length and 324 µm in diameter, which exhibited a high spectral resolution, characterized by a narrow linewidth of merely 0.9 µm.^[^ ^317^ ^]^ Researchers developed a flexible thin‐film electrode ≈8 µm thick using printed electronics for electrocorticography recording and electrical stimulation.^[^ ^318^ ^]^ The fabrication process involved inkjet printing gold nanoink onto a polystyrene‐block‐polybutadiene‐block‐polystyrene (SBS) substrate, followed by encapsulation with another SBS thin film as insulation. The electrode, with 35 channels, was implanted on the cortical surface of rats and demonstrated successful electrocorticography recording and stimulation without significant inflammatory reactions after 6 weeks. Notably, the electrode effectively visualized epileptiform activity in a drug‐induced epilepsy rat model, highlighting its potential for managing intractable epilepsy.^[^ ^318^ ^]^ Another proof of concept is the development of an inkjet‐printed, millimeter‐scale system on a chip for wirelessly powered bidirectional neural communication, integrating optogenetic stimulation and electrophysiological recording capabilities.^[^ ^319^ ^]^ The incorporation of inkjet‐printed microlenses improved light directivity, and a low‐noise two‐stage recording front‐end facilitated simultaneous monitoring of neural activity. The system on a chip's efficacy was demonstrated by its ability to activate channelrhodopsin (hChR_2_) in mouse Neuro2a cells, resulting in a twofold increase in intracellular calcium levels.^[^ ^319^ ^]^ Additionally, researchers developed an ultra‐small neural stimulating implant (≈0.009 mm^3^) using inkjet printing and 130 nm CMOS technology.^[^ ^320^ ^]^ This fully encapsulated microbead, measuring 200 µm × 200 µm × 80 µm, integrates wireless powering, microelectronics, electrodes, and a coil. Minimally invasive implantation into the sciatic nerve of a rat model demonstrated successful elicitation of compound action potentials, validating the implant's functionality for potential central nervous system applications. Additionally, a recording microbead with wireless powering and backscattering telemetry was investigated, showcasing the versatility of this technology for future neural interfaces.^[^ ^320^ ^]^ Although 3D inkjet printing has yet to be explored for fabricating coils in magnetic neurostimulation, its scalability, noncontact nature, and adaptability to diverse materials make it a promising avenue for future research.

##### Electrohydrodynamic Printing

Compared to inkjet printing, electrohydrodynamic printing offers a higher resolution,^[^ ^323^ ^]^ which is attained by using a droplet formation approach involving the usage of an electric field between the substrate and miniature nozzles, typically with inner diameters ranging from about 100 nm to several micrometers.^[^ ^321^, ^324^ ^]^ In this process, the ink is transported to the nozzle, and the application of an electric field causes a buildup of mobile ions at the nozzle tip on the ink's surface. This results in electromagnetic “Maxwell” stresses at the nozzle tip, leading to the formation of a Taylor cone. When the electric field reaches a certain threshold, the stresses at the nozzle tip can surpass the opposing capillary stresses. Based on the electrical characteristics of the ink this can result in the ejection from the nozzle as a i) fine jet or droplets ii) also known as electrohydrodynamic jet printing or electrohydrodynamic nanodrip printing, respectively,^[^ ^321^, ^325^ ^]^ like presented in Figure 8c. It has been shown that these methods have the capability of printing various materials with a precision of nanometer on both structured and flat substrates such as colloidal quantum dots,^[^ ^326^ ^]^ nanoparticles,^[^ ^327^ ^]^ silver and gold,^[^ ^328^ ^]^ and polymers.^[^ ^329^ ^]^ To demonstrate the feasibility of electrohydrodynamic printing for coil fabrication, a spiral‐type inductor was printed.^[^ ^330^ ^]^ The printed inductor, exhibiting an inductance of 9.45 µH, displayed approximately fivefold higher resistivity compared to bulk silver following sintering. This method also has been employed to print a silver‐based spiral inductor with micrometer resolution.^[^ ^307^ ^]^ Thus, the electrohydrodynamic printing technique offers a promising avenue for fabricating microscale electronic devices, primarily due to its capability to produce high‐resolution features and its versatility in accommodating a wide variety of functional inks.^[^ ^331^ ^]^ As further evidence of the potential of this technology, researchers have demonstrated the successful printing of various passive electrical components, including capacitors, resistors, and inductors, with a minimum feature size of ≈60 µm, achieved using a 110 µm nozzle.^[^ ^307^ ^]^ This highlights the potential of electrohydrodynamic printing for the fabrication of complex electronic devices.^[^ ^332^ ^]^ While it has been demonstrated that inks with a range of electrical conductivities can be printed using this method, the necessity for an electric field imposes certain limitations on the process concerning the characteristics of the ink and substrate compatibility.^[^ ^333^ ^]^ For example, using a DC voltage the initiation and cessation of liquid jetting cannot be precisely controlled. Additionally, when a pulse voltage is employed there exists a nonlinear relationship between the frequency of droplet deposition and applied voltage. Both scenarios affect the fine resolution of the printed structures.^[^ ^334^ ^]^ Currently, this potential application underscores the transformative possibilities of electrohydrodynamic printing, not only for electronics manufacturing but for neuromodulation therapies as well.^[^ ^335^, ^336^, ^337^ ^]^ Further investigation into this synergy between electrohydrodynamic printing and magnetic neurostimulation coil fabrication is thus warranted, as it could open doors to novel treatment modalities and enhanced therapeutic outcomes.

##### Aerosol Jet Printing

Aerosol jet printing provides an enhanced line resolution, achieving widths as fine as 10 µm, which is an improvement over the capabilities of inkjet printing.^[^ ^338^ ^]^ In this method, an ink containing the desired nanoparticles is subjected to ultrasonic atomization, creating a dense slurry of ink droplets with diameters ranging approximately from 1 to 5 µm. The size of the droplets in this process is dependent on the atomizer frequency.^[^ ^322^, ^339^ ^]^ As Figure 8d shows, an inert gas is used to carry the aerosolized droplets from the reservoir of the ink to the printhead. Within the printhead, a sheath gas stream collimates and speeds up the flow of microdroplets in a tapered nozzle. This action induces aerodynamic focusing, forming a precise jet that affects the substrate surface.^[^ ^339^ ^]^ The independence of the deposition to the direction and ample spacing between the substrate and printer tip enable consistent material deposition on uneven and irregular surfaces, making it ideal for printing on nonplanar substrates conformally.^[^ ^340^, ^341^ ^]^ This printing technique is capable of depositing not only metallic nanoparticles but also a diverse range of materials, such as 2D substances (e.g., graphene) and thermoelectric.^[^ ^340^, ^341^ ^]^ As a preliminary step toward realizing the potential of aerosol jet printing for microcoil array fabrication, silver nanoparticle‐based human‐sized coils were successfully prototyped using this technology.^[^ ^308^ ^]^ These reproducibly fabricated coils demonstrated a diameter of 1800 µm, trace width and spacing of 112.5 µm, a thickness of 12 µm, and an inductance value of ≈15.5 nH. Modeling data revealed that the coils generated a confined depolarization–hyperpolarization region of 1.5 mm, suggesting a more focused activation pattern compared to traditional cochlear implants.^[^ ^308^ ^]^ Another attempt focused on the process and characterization of printed inductors of polymer‐matrix magnetic nanocomposites, comprising nickel‐zinc ferrite (Ni_0.5_Zn_0.5_Fe_2_O_4_) nanoparticles in a polyimide matrix using multimaterial aerosol jet printing. The results showed a 40% increase in inductance density in 1‐mm^2^, 4.5‐turn microstrip spiral inductors printed with the composite, achieving an inductance density of 4.2 nH mm^−^^2^. Energy‐dispersive X‐ray spectroscopy analysis revealed films with 18 wt% Ni_0.5_Zn_0.5_Fe_2_O_4_. Additionally, microstrip transmission lines on the printed composite exhibited magnetic resonance behavior when subjected to a DC magnetic bias.^[^ ^342^ ^]^ In a demonstration of aerosol‐jet printing's versatility, high‐frequency, tapered‐solenoid inductors with wide bandwidth capabilities were successfully fabricated.^[^ ^343^ ^]^ The design strategically incorporated a polymer support structure to minimize parasitic capacitance, a tapered solid core, and conductive windings. Two variants were produced, one featuring a printed polymer core and the other utilizing a nonprinted iron core. Scattering parameter analysis confirmed that the polymer‐core inductor achieved a usable bandwidth of up to 18 GHz, while the iron‐core inductor extended to 40 GHz, both maintaining low insertion loss.^[^ ^343^ ^]^ Additionally, researchers demonstrated the fabrication of a coreless flyback transformer using aerosol jet printing and electrodeposition techniques.^[^ ^344^ ^]^ A two‐layer secondary inductor was built on top of a two‐layer primary inductor, separated by UV‐curable dielectric polymer layers. The transformer achieved a gain of 75.3x, converting a 17 V, 400 kHz input into a 1250 V output. Additionally, the study showcased the successful fabrication of 8‐layer stacked inductors with low equivalent series resistance (0.6 Ω) and 1.7 µH inductance at 100 kHz.^[^ ^344^ ^]^ However, despite the capability to deposit various kinds of inks, setting the optimal printing boundaries is often challenging. For example, the rheology of the employed ink is a crucial factor in this technique, determining the jet‐ability of the ink and affecting the printing quality.^[^ ^345^ ^]^ Achieving reproducibility and consistency is another challenge, often necessitating rigorous steps like ink replacement several times during the process, which can impede the scalability of the production.^[^ ^346^, ^347^, ^348^ ^]^ Despite these challenges, the precision, scalability, and versatility inherent to this method suggest its capacity to address the intricate demands of this field and facilitate the development of innovative neurostimulation devices.

### 4.4 Overview of Microfabrication Technologies

In conclusion, the diverse fabrication methods explored to produce coils for magnetic neurostimulation each offer unique advantages and limitations (**Table** **4**). TSVs technology enables compact, high‐density coils with embedded windings and integrated cores, but faces challenges in fragility and complex fabrication processes. Surface micromachining allows for precise control over coil geometry and offers versatility in creating various coil shapes but can be limited by substrate interactions and complexity in 3D fabrication. Lithography techniques provide high resolution and the ability to create complex patterns yet may be limited by cost and processing speed for certain methods. FEBID excels in depositing high‐resolution, complex 3D structures, but is currently limited by throughput and material constraints. 3D printing technologies like aerosol jet printing offer versatile material compatibility and conformal printing on nonplanar substrates but require optimization of printing parameters for reproducibility. Screen printing and inkjet printing provide simple, scalable, and cost‐effective solutions, but with lower resolution compared to other techniques. Electrohydrodynamic printing offers high resolution and versatility but faces challenges in parameter optimization and reproducibility. Overall, the choice of fabrication method depends on the specific requirements of the neurostimulation application, balancing factors such as coil size, geometry, performance, and cost. Continued research and development in these areas will undoubtedly lead to further advancements in the field of magnetic neurostimulation, ultimately benefiting patients and expanding the therapeutic potential of this technology.

**Table 4:** Summary of the key characteristics and comparison of microfabrication technologies.

Fabrication method	Geometry	Applications in Neurostimulation	Physical dimensions	Key characteristics	Limitations	Refs
Through‐Substrate Vias	Spiral/ Toroidal solenoid	DBS/ cortical stimulation/ peripheral nerve stimulation/ selective activation	100s nm to 100s µm	Compact spiral windings embedded in substrate and core/ high aspect ratio/ free of voids/ substrate isolation by suspended windings.	Fragile coils/ complex process	[222, 224, 225, 226, 227, 232, 349, 350, 351, 352, 353, 354, 355, 356, 357]
2D/3D micromachining	Spiral/ Toroidal/ Racetrack solenoid/ planar coils	Cortical stimulation/ peripheral nerve stimulation/ selective activation	100 nm to 100s µm	High precision/ CMOS compatible	Low quality factor (2D)/ eddy currents/ the complex process (3D)	[358, 359, 360, 361, 362]
Lithography/ Two‐photon polymerization	Microprobes/ planar coils	DBS/ cortical stimulation/ peripheral nerve stimulation	10s nm to 100 µm	High precision	Wavelength limitation to size/ low processing speed	[259, 272, 275, 283, 363]
Focused electron beam‐induced deposition	Helical nanostructure	Magnetic force microscopy/ nanoscale coils	Sub‐Å to 10s nm	High resolution/ direct deposition/ in‐situ inspection/ compatible with nonflat substrates/ 3D structure efficient patterning	Low throughput/ conductive material limitation/ slow speed	[284, 285, 287, 293, 302, 303]
Screen printing	Microelectrode	Cortical stimulation/ wearable electronics	50‐100 µm to large	Simple/ scalable/ cost‐effective	Lowe resolution	[310, 312]
Inkjet printing	Solenoid/ Implantable microprobes	Implants/ flexible electronics	20 to 100s µm	Low pressure and temperature/ multi‐material capability/ technological flexibility/ scalable	Limited resolution and shape range	[316, 317, 318, 320]
Electrohydrodynamic printing	Spiral solenoid	Microscale complex magnetic electronics	100 nm to 1–10 µm	High resolution/ multi‐material compatibility/ compatible with nonplanar substrates	Challenges in parameter optimization/ reproducibility/ consistency/ equipment access/ ink, substrate compatibility/ conductivity	[330, 333]
Aerosol jet printing	Spiral solenoid	Cochlear implants/ high‐frequency complex magnetic electronics	10 to 100s μm	High resolution/ versatile material compatibility/ conformal printing on nonplanar substrates	Challenges in parameter optimization/ reproducibility/ consistency	[308, 338, 343, 345, 364, 365, 366]

## 5 Conclusion and Future Work

### 5.1 Noninvasive Magnetic Stimulation

The noninvasive method relies on Faraday's law to transmit stimulation signals by modulating magnetic fields. TMS stands as one of the earliest magnetic neuromodulation techniques and has thus matured into a well‐established and widely used method. It benefits from extensive clinical experience. The primary advantage of TMS is its “noninvasive” nature, eliminating the risks associated with implantation, such as immune responses.

However, the noninvasive feature of TMS also poses certain challenges. Firstly, its focality has historically been less precise, although this has improved over time with the development of advanced coil designs, as discussed earlier. Secondly, TMS struggles to deliver robust stimulation to deep brain regions. Although the introduction of the H‐coil has mitigated this limitation to some extent, DBS remains challenging for TMS. Thirdly, TMS applies stimulation to the entire head, making it difficult to achieve precise targeting of specific neurons or lesions. This results in lower selectivity and resolution. Consequently, TMS cannot provide highly accurate neuromodulation, and despite advancements in brain mapping, a definitive solution to this issue remains elusive.

The potential of TMS appears to be reaching a stable stage. While ongoing research in brain mapping and stimulation waveforms continues to yield incremental progress for TMS, it is unlikely to bring about fundamental changes in the technology. Although coil design remains a potential avenue for improvement, the pace of innovation in this regard has been slowing down. It is challenging to make precise predictions of TMS. However, it's essential to acknowledge that TMS has amassed a wealth of technical experience in coil design and a substantial clinical database. These resources can be leveraged in the development of next‐generation magnetic neuromodulation techniques, such as nanoinvasive magnetic neuromodulation coil designs. The convergence of this accumulated knowledge with emerging technologies may lead to breakthroughs in the field of magnetic neuromodulation.

### 5.2 Invasive Magnetic Neuromodulation

The invasive application of Faraday's law at a microscale represents the primary approach of µMS. Its proximity to the targeted lesion significantly enhances stimulation resolution, enabling the feasibility of magnetic DBS. The induced electric field in µMS is both computable and more controllable. When combined with µMS's spatial selectivity, it allows for highly precise stimulation, particularly when targeting neurons with distinct spatial distributions. Additionally, µMS is advantageous due to its relatively low current requirements for stimulation, contributing to improved power efficiency. These features collectively position µMS as a promising technique for highly accurate and localized neural stimulation, particularly in scenarios demanding precise spatial targeting and chronic implantation.

A notable drawback of it is invasiveness, which might bring extra risks from surgery or inflammation. However, µMS, compared with other invasive methods as a magnetic neuromodulation approach, the coil does not contact tissue directly. The microcoil, if encapsulated within biocompatible materials, can serve a dual purpose: protecting surrounding tissue from potential immune responses and other implant‐related side effects while safeguarding the coil from degradation due to the tissue environment. This encapsulation supports the utilization of certain non‐biocompatible materials in the fabrication of the coil.

µMS exhibits remarkable qualities such as precision, selectivity, compactness, low power requirements, and the potential for chronic implantation. In the short term, its high precision and selectivity for neuromodulation make it a valuable tool for advancing neuroscience research. This research experience can further inform and refine its applications. Looking to the long term, the additional attributes of µMS align well with the characteristics of mobile medical devices. Consequently, µMS could serve as a method for neuromodulation in telemedicine or at‐home medical settings. However, it is essential to acknowledge that this technological avenue is still in its early stages and lacks extensive clinical experience. As an invasive technology, to realize its potential, concerted efforts should focus on in vivo testing to assess the safety and efficacy of invasive neuromodulation therapy. Additionally, system integration plays a pivotal role in bridging the gap between this emerging technology and its practical application in individuals' homes.

### 5.3 Nanoinvasive Magnetic Neuromodulation

The nanoinvasive method harnesses magnetic fields to manipulate nanoparticles for neuromodulation, representing a delicate balance between invasive and noninvasive approaches. The absence of mechanical implantation like what µMS does, which will significantly reduce the risk of side effects brought by implantation, and its high stimulation selectivity derived from specific ligand‐receptor interactions can ensure accurate selection of stimulation target or stimulation mode (activation or inhibition). Moreover, the biomolecules (ion channels) associated with thermal and mechanical methods are more widely distributed within humans, mitigating ethical concerns associated with genetic modifications.

These compelling advantages come at a cost: the process is marked by the complexity of particle coating and delivery to the targeted tissue. Achieving the requisite level of selectivity necessitates a meticulously designed particle coating to enable specific binding to receptors. However, the applications of MNPs in other areas like cancer treatment have accumulated rich experience for nanoinvasive magnetic neuromodulation, which aids in the translation of this neuromodulation approach.

At present, the challenges in the field of nanoparticle‐based neuromodulation include addressing the issues related to targeting and delivery of nanoparticles, for example, crossing the blood‐brain barrier. One of the most significant gaps between the technology and practical application lies in the lack of suitable coils. It is imperative to design the miniaturized and wearable coil specifically for nanoinvasive magnetic neuromodulation. Although, like µMS, clinical application is still not performed, with more in vitro/in vivo experiments and further suitable coil designs, this technology has the potential to lead the next‐generation magnetic neuromodulation.

### 5.4 Microfabrication Strategies

The manufacturing of microcoils for magnetic stimulation presents both challenges and exciting opportunities in advanced medical technologies. Achieving miniaturization is a significant challenge, as a crucial aspect of convenient, portable, and chronic magnetic neurostimulation. The fabrication processes of microcoils, whether TSVs, surface micromachining, or emerging techniques like 3D micro/nanofabrication techniques, require meticulous consideration of materials, size, and operational properties.

Successful applications of microfabrication have been demonstrated in microprobes, microsolenoids, and planar coils, showcasing the feasibility of microcoils in neuromodulation and the vast possibilities they offer. However, as repeatedly emphasized, there is limited experience in applying microfabrication to neural modulation, particularly in nanoinvasive neuromodulation coils, which requires further evidence to prove its applicability. According to Section 4, most existing mini‐/microcoil applications are in the realm of µMS. Therefore, more focus should be placed on mini‐/microcoils for nanoinvasive magnetic neuromodulation. Although, certain technologies, like screen printing, remain unexplored in this field. With their biocompatibility and ability to fabricate complex structures, these techniques are worth exploring for neuromodulation coil fabrication.

TSVs offer the flexibility to design coils with dimensions spanning from sub‐micrometers to several hundred micrometers. Similarly, surface micromachining has proven effective in crafting microcoils within a comparable size range with higher precision. However, it is important to note that these multistep methods, while successful, are time‐consuming, and the resultant coils are vulnerable to damage and fragility. TPS enables the fabrication of nanoscale structures with superior flexibility and resolution, but its processing speed and occurrence of intralayer surfaces present challenges. FEBID showcases capabilities for depositing materials at micro‐ and nanometer scales, allowing for intricate 3D nanoconstruct patterning, but challenges exist in achieving higher throughput for neurostimulation applications. Alternatively, rapid and cost‐effective methods such as screen printing, inkjet printing, electrohydrodynamic printing, and aerosol jet printing offer scalability and flexibility. Each method has its strengths and limitations, with considerations ranging from resolution and material compatibility to reproducibility and deposition challenges. Yet to drive ongoing progress, challenges persist in achieving the cost‐effective, mass production of high‐performance miniature microcoils. Future trends in microcoil design, particularly for magnetic neurostimulation applications, necessitate the use of flexible substrates. Consequently, it is essential to devise more appropriate techniques for the integration of the substrate with materials. With a miniature coil size and elevated levels of integration, the corresponding rise in working temperature will be observed. In the case of most insulating compounds, with rising temperatures their resistance tends to decrease exponentially, leading to a significant decline in insulation performance. So, it is imperative to have insulating materials with enhanced stability and temperature resistance such as a composite of insulating layers. As the field evolves, future perspectives may involve refining these techniques to address current limitations and advancing toward more efficient and scalable microcoil manufacturing.

## Conflict of Interest

The authors declare no conflict of interest.

## References

[1] J. M. Schwalb , C. Hamani , Neurotherapeutics 2008, 5, 3. 18164479 10.1016/j.nurt.2007.11.003 PMC5084122

[2] K. Starnes , K. Miller , L. Wong‐Kisiel , B. N. Lundstrom , Brain sciences 2019, 9, 283. 31635298 10.3390/brainsci9100283 PMC6826633

[3] S. L. Tripathi , K. B. Prakash , V. E. Balas , S. K. Mohapatra , J. Nayak , Electronic Devices, Circuits, and Systems for Biomedical Applications: Challenges and Intelligent Approach, Academic Press, London, UK 2021.

[4] D. Magis , J. Schoenen , Lancet Neurol. 2012, 11, 708. 22814542 10.1016/S1474-4422(12)70139-4

[5] W. Schuepbach , J. Rau , K. Knudsen , J. Volkmann , P. Krack , L. Timmermann , T. Hälbig , H. Hesekamp , S. Navarro , N. Meier , N. Engl. J. Med. 2013, 368, 610. 23406026 10.1056/NEJMoa1205158

[6] G. K. Bergey , Exp. Neurol. 2013, 244, 87. 23583414 10.1016/j.expneurol.2013.04.004

[7] O. World Health , Neurological Disorders: Public Health Challenges, World Health Organization, Geneva 2006.

[8] S. F. Cogan , K. A. Ludwig , C. G. Welle , P. Takmakov , J. Neural Eng. 2016, 13, 021001. 26792176 10.1088/1741-2560/13/2/021001 PMC5386002

[9] A. Butterwick , A. Vankov , P. Huie , Y. Freyvert , D. Palanker , IEEE Trans. Biomed. Eng. 2007, 54, 2261. 18075042 10.1109/tbme.2007.908310

[10] A. C. Thompson , P. R. Stoddart , E. D. Jansen , Curr. Mol. Imaging (Discontin.) 2014, 3, 162. 10.2174/2211555203666141117220611 PMC4541079 26322269

[11] J. A. Cardin , M. Carlén , K. Meletis , U. Knoblich , F. Zhang , K. Deisseroth , L.‐H. Tsai , C. I. Moore , Nat. Protoc. 2010, 5, 247. 20134425 10.1038/nprot.2009.228 PMC3655719

[12] F. Gilbert , A. R. Harris , R. M. Kapsa , AJOB Neuroscience 2014, 5, 3.

[13] R. S. Weiss , A. Voss , W. Hemmert , Netw.: Comput. Neural Syst. 2016, 27, 212. 10.1080/0954898X.2016.1224944 27644125

[14] Y. Tufail , A. Matyushov , N. Baldwin , M. L. Tauchmann , J. Georges , A. Yoshihiro , S. I. H. Tillery , W. J. Tyler , Neuron 2010, 66, 681. 20547127 10.1016/j.neuron.2010.05.008

[15] G. Darmani , T. Bergmann , K. B. Pauly , C. Caskey , L. De Lecea , A. Fomenko , E. Fouragnan , W. Legon , K. Murphy , T. Nandi , Clin. Neurophysiol. 2022, 135, 51. 35033772 10.1016/j.clinph.2021.12.010

[16] P. M. Rossini , D. Burke , R. Chen , L. Cohen , Z. Daskalakis , R. Di Iorio , V. Di Lazzaro , F. Ferreri , P. Fitzgerald , M. George , Clin. Neurophysiol. 2015, 126, 1071. 25797650 10.1016/j.clinph.2015.02.001 PMC6350257

[17] P. J. Basser , B. J. Roth , Annu. Rev. Biomed. Eng. 2000, 2, 377. 11701517 10.1146/annurev.bioeng.2.1.377

[18] A. V. Peterchev , T. A. Wagner , P. C. Miranda , M. A. Nitsche , W. Paulus , S. H. Lisanby , A. Pascual‐Leone , M. Bikson , Brain Stimul. 2012, 5, 435. 22305345 10.1016/j.brs.2011.10.001 PMC3346863

[19] S. W. Lee , K. Thyagarajan , S. I. Fried , IEEE Trans. Biomed. Eng. 2019, 66, 1680. 30369434 10.1109/TBME.2018.2877713 PMC6561646

[20] R. Chen , G. Romero , M. G. Christiansen , A. Mohr , P. Anikeeva , Science 2015, 347, 1477. 25765068 10.1126/science.1261821

[21] M. d'Arsonval , Comput. rend. Soc. biol. 1896, 3, 430.

[22] L. A. Geddes , IEEE Eng. Med. Biol. Mag. 2008, 27, 101. 10.1109/MEMB.2007.911401 18270053

[23] M. Polson , Med. Biol. Eng. Comput. 1982, 20, 243. 7098583 10.1007/BF02441362

[24] A. T. Barker , R. Jalinous , I. L. Freeston , Lancet 1985, 325, 1106. 10.1016/s0140-6736(85)92413-4 2860322

[25] Y. Terao , Y. Ugawa , J. Clin. Neurophysiol. 2002, 19, 322. 12436088 10.1097/00004691-200208000-00006

[26] M. Hallett , Neuron 2007, 55, 187. 17640522 10.1016/j.neuron.2007.06.026

[27] X. Yang , E. McGlynn , R. Das , S. P. Paşca , B. Cui , H. Heidari , Adv. Mater. 2021, 33, 2103208. 34668249 10.1002/adma.202103208 PMC8712412

[28] A. Barker , I. Freeston , R. Jalinous , J. Jarratt , Neurosurgery 1987, 20, 100. 3808249 10.1097/00006123-198701000-00024

[29] D. Cappon , T. den Boer , C. Jordan , W. Yu , E. Metzger , A. Pascual‐Leone , Ageing Res. Rev. 2022, 74, 101531. 34839043 10.1016/j.arr.2021.101531 PMC8996329

[30] X. Jiang , W. Yan , R. Wan , Y. Lin , X. Zhu , G. Song , K. Zheng , Y. Wang , X. Wang , Neurosci. Biobehav. Rev. 2022, 132, 130. 34826512 10.1016/j.neubiorev.2021.11.037

[31] H. Tavakoli , A. Heidarpanah , Iranian J. Child Neurol. 2023, 17, 9. 10.22037/ijcn.v17i2.38752 PMC9881833 36721826

[32] F. A. Somaa , T. A. de Graaf , A. T. Sack , Front. Neurol. 2022, 13, 793253. 35669870 10.3389/fneur.2022.793253 PMC9163300

[33] X. Zong , J. Gu , D. Geng , D. Gao , Neurochem. Int. 2022, 157, 105356. 35513205 10.1016/j.neuint.2022.105356

[34] O. Numssen , A.‐L. Zier , A. Thielscher , G. Hartwigsen , T. R. Knösche , K. Weise , NeuroImage 245, 118654. 10.1016/j.neuroimage.2021.118654 34653612

[35] S. Minusa , T. Tateno , Int. Conf. Neural Information Processing 2016.

[36] H. Tischler , S. Wolfus , A. Friedman , E. Perel , T. Pashut , M. Lavidor , A. Korngreen , Y. Yeshurun , I. Bar‐Gad , J. Neurosci. Methods 2011, 194, 242. 20974177 10.1016/j.jneumeth.2010.10.015

[37] G. Bonmassar , S. W. Lee , D. K. Freeman , M. Polasek , S. I. Fried , J. T. Gale , Nat. Commun. 2012, 3, 2012. 10.1038/ncomms1914 PMC3621430 22735449

[38] H.‐J. Park , G. Bonmassar , J. A. Kaltenbach , A. G. Machado , N. F. Manzoor , J. T. Gale , Nat. Commun. 2013, 4, 2463. 24030203 10.1038/ncomms3463 PMC3845906

[39] S. W. Lee , S. I. Fried , IEEE Trans. Neural Syst. Rehabil. Eng. 2014, 23, 116. 25163063 10.1109/TNSRE.2014.2348415 PMC4467829

[40] S. Mukesh , D. Blake , B. McKinnon , P. Bhatti , IEEE Trans. Neural Syst. Rehabil. Eng. 2016, 25, 1353. 27831887 10.1109/TNSRE.2016.2624275 PMC5750049

[41] S. W. Lee , F. Fallegger , B. D. F. Casse , S. I. Fried , Sci. Adv. 2016, 2, e1600889. 27957537 10.1126/sciadv.1600889 PMC5148213

[42] G. E. Salvi , U. Brägger , Int. J. Oral Maxillofac. Implants 2009, 24 Suppl, 69. 19885435

[43] J. B. Weaver , A. M. Rauwerdink , E. W. Hansen , Med. Phys. 2009, 36, 1822. 19544801 10.1118/1.3106342 PMC4109636

[44] S. Mornet , S. Vasseur , F. Grasset , P. Veverka , G. Goglio , A. Demourgues , J. Portier , E. Pollert , E. Duguet , Prog. Solid State Chem. 2006, 34, 237.

[45] R. Hergt , S. Dutz , R. Müller , M. Zeisberger , J. Phys.: Condens. Matter 2006, 18, S2919.

[46] I. Šafařík , M. Šafaříková , Magnetic Nanoparticles and Biosciences, Springer, Vienna, Austria 2002.

[47] Q. A. Pankhurst , J. Connolly , S. K. Jones , J. Dobson , J. Phys. D: Appl. Phys. 2003, 36, R167.

[48] M. M. Selim , S. El‐Safty , A. Tounsi , M. Shenashen , APL Mater 2024, 12, 010601.

[49] H. Huang , S. Delikanli , H. Zeng , D. M. Ferkey , A. Pralle , Nat. Nanotechnol. 2010, 5, 602. 20581833 10.1038/nnano.2010.125

[50] J.‐H. Lee , J.‐w. Kim , M. Levy , A. Kao , S.‐h. Noh , D. Bozovic , J. Cheon , ACS Nano 2014, 8, 6590. 25004005 10.1021/nn5020616

[51] J.‐w. Kim , H.‐k. Jeong , K. M. Southard , Y.‐w. Jun , J. Cheon , Acc. Chem. Res. 2018, 51, 839. 29589897 10.1021/acs.accounts.8b00004 PMC5917604

[52] S. Hughes , A. J. El Haj , J. Dobson , Med. Eng. Phys. 2005, 27, 754. 15985383 10.1016/j.medengphy.2005.04.006

[53] S. Hughes , S. McBain , J. Dobson , A. J. El Haj , J. R. Soc., Interface 2008, 5, 855. 18077244 10.1098/rsif.2007.1274 PMC2495030

[54] B. Coste , J. Mathur , M. Schmidt , T. J. Earley , S. Ranade , M. J. Petrus , A. E. Dubin , A. Patapoutian , Science 2010, 330, 55. 20813920 10.1126/science.1193270 PMC3062430

[55] S. A. Stanley , J. E. Gagner , S. Damanpour , M. Yoshida , J. S. Dordick , J. M. Friedman , Science 2012, 336, 604. 22556257 10.1126/science.1216753 PMC3646550

[56] E. A. Périgo , G. Hemery , O. Sandre , D. Ortega , E. Garaio , F. Plazaola , F. J. Teran , Appl. Phys. Rev. 2015, 2, 041302.

[57] M. G. Christiansen , A. Senko , R. Chen , G. Romero , P. Anikeeva , Appl. Phys. Lett. 2014, 104.

[58] D. Gregurec , A. W. Senko , A. Chuvilin , P. D. Reddy , A. Sankararaman , D. Rosenfeld , P.‐H. Chiang , F. Garcia , I. Tafel , G. Varnavides , ACS Nano 2020, 14, 8036. 32559057 10.1021/acsnano.0c00562 PMC8592276

[59] D. Rosenfeld , A. W. Senko , J. Moon , I. Yick , G. Varnavides , D. Gregureć , F. Koehler , P.‐H. Chiang , M. G. Christiansen , L. Y. Maeng , Sci. Adv. 2020, 6, eaaz3734. 32300655 10.1126/sciadv.aaz3734 PMC7148104

[60] S.‐A. Hescham , P.‐H. Chiang , D. Gregurec , J. Moon , M. G. Christiansen , A. Jahanshahi , H. Liu , D. Rosenfeld , A. Pralle , P. Anikeeva , Nat. Commun. 2021, 12, 5569. 34552093 10.1038/s41467-021-25837-4 PMC8458499

[61] C. Sebesta , D. T. Hinojosa , B. Wang , J. Asfouri , Z. Li , G. Duret , K. Jiang , Z. Xiao , L. Zhang , Q. Zhang , V. L. Colvin , S. M. Goetz , A. V. Peterchev , H. A. Dierick , G. Bao , J. T. Robinson , Nat. Mater. 2022, 21, 951. 35761060 10.1038/s41563-022-01281-7 PMC10965118

[62] Deep Brain Magnetothermal Silencing of Dopaminergic Neurons via Endogenous TREK1 Channels Abolishes Place Preference in Mice , NewsRX LLC, 2024, p. 1063.

[63] L. Sapir , S. Tzlil , Semin. Cell Devel. Biol. 2017, 71, 99. 28630027 10.1016/j.semcdb.2017.06.010

[64] M. Kobayashi , A. Pascual‐Leone , Lancet Neurol. 2003, 2, 145. 12849236 10.1016/s1474-4422(03)00321-1

[65] A. Rotenberg , J. C. Horvath , A. Pascual‐Leone , in Springer, 2014.

[66] A. H. Iglesias , Curr. Neurol. Neurosci. Rep. 2020, 20, 1. 32020300 10.1007/s11910-020-1021-0

[67] B. He , Neural Engineering, Springer, Cham, Switzerland 2005.

[68] J. Selvaraj , P. Rastogi , N. Prabhu Gaunkar , R. L. Hadimani , M. Mina , IEEE Trans. Magn. 2018, 54, 5200405.

[69] V. Walsh , M. Rushworth , Neuropsychologia 1999. 10080370

[70] B. Guse , P. Falkai , T. Wobrock , J. Neural Transm. 2010, 117, 105. 19859782 10.1007/s00702-009-0333-7 PMC3085788

[71] A. Pascual‐Leone , A. Dhuna , B. Roth , L. Cohen , M. Hallett , Lancet 1990, 336, 1195. 10.1016/0140-6736(90)92815-y 1978057

[72] B. J. Roth , A. Pascual‐Leone , L. G. Cohen , M. Hallett , Electroencephalogr. Clin. Neurophysiol./Evoked Potentials Sect. 1992, 85, 116. 10.1016/0168-5597(92)90077-o 1373364

[73] L. S. Pereira , V. T. Müller , M. da Mota Gomes , A. Rotenberg , F. Fregni , Epilepsy Behav. 2016, 57, 167. 26970993 10.1016/j.yebeh.2016.01.015

[74] A. Hufnagel , D. Clause , C. Brunhoelzl , T. Sudhop , J. Neurol. 1993, 240, 373. 8336179 10.1007/BF00839970

[75] A. Pascual‐Leone , C. Houser , K. Reese , L. Shotland , J. Grafman , S. Sato , J. Valls‐Sole , J. Brasil‐Neto , E. Wassermann , L. Cohen , Electroencephalogr. Clin. Neurophysiol./Evoked Potent. Sect. 1993, 89, 120. 10.1016/0168-5597(93)90094-6 7683602

[76] J.‐P. Lefaucheur , N. André‐Obadia , A. Antal , S. S. Ayache , C. Baeken , D. H. Benninger , R. M. Cantello , M. Cincotta , M. de Carvalho , D. De Ridder , Clin. Neurophysiol. 2014, 125, 2150. 25034472 10.1016/j.clinph.2014.05.021

[77] J.‐P. Lefaucheur , A. Aleman , C. Baeken , D. H. Benninger , J. Brunelin , V. Di Lazzaro , S. R. Filipović , C. Grefkes , A. Hasan , F. C. Hummel , Clin. Neurophysiol. 2020, 131, 474. 31901449 10.1016/j.clinph.2019.11.002

[78] X. Che , R. F. Cash , X. Luo , H. Luo , X. Lu , F. Xu , Y.‐F. Zang , P. B. Fitzgerald , B. M. Fitzgibbon , Brain Stimul. 2021, 14, 1135. 34280583 10.1016/j.brs.2021.07.004

[79] G. Pateraki , K. Anargyros , A.‐M. Aloizou , V. Siokas , C. Bakirtzis , I. Liampas , Z. Tsouris , P. Ziogka , M. Sgantzos , V. Folia , J. Electromyogr. Kinesiol. 2022, 62, 102622. 34890834 10.1016/j.jelekin.2021.102622

[80] J. Zhang , D. Zhong , X. Xiao , L. Yuan , Y. Li , Y. Zheng , J. Li , T. Liu , R. Jin , Clin. Rehab. 2021, 35, 1103. 10.1177/0269215521999554 33706572

[81] M. Sommer , A. Alfaro , M. Rummel , S. Speck , N. Lang , T. Tings , W. Paulus , Clin. Neurophysiol. 2006, 117, 838. 16495145 10.1016/j.clinph.2005.10.029

[82] K. Wendt , M. M. Sorkhabi , C. J. Stagg , M. K. Fleming , T. Denison , J. O'Shea , Brain Stimul. 2023, 16, 1178. 37543172 10.1016/j.brs.2023.08.001 PMC10444700

[83] N. Arai , S. Okabe , T. Furubayashi , Y. Terao , K. Yuasa , Y. Ugawa , Clin. Neurophysiol. 2005, 116, 605. 15721074 10.1016/j.clinph.2004.09.020

[84] K. Rösler , C. Hess , R. Heckmann , H. Ludin , Neurosci. Lett. 1989, 100, 347. 2761784 10.1016/0304-3940(89)90711-8

[85] S. Ueno , T. Tashiro , K. Harada , J. Appl. Phys. 1988, 64, 5862.

[86] L. G. Cohen , B. J. Roth , J. Nilsson , N. Dang , M. Panizza , S. Bandinelli , W. Friauf , M. Hallett , Electroencephalogr. Clin. Neurophysiol. 1990, 75, 350. 1691084 10.1016/0013-4694(90)90113-x

[87] D. Cohen , B. N. Cuffin , J. Clin. Neurophysiology 1991, 8, 102. 10.1097/00004691-199101000-00013 2019645

[88] K. Yunokuchi , D. Cohen , J. Clin. Neurophysiol.: Off. Publ. Am. Electroencephalogr. Soc. 1991, 8, 112. 2019646

[89] G. G. Westin , B. D. Bassi , S. H. Lisanby , B. Luber , N. U. New York State Psychiatric Institute , Clin. Neurophysiol. 2014, 125, 142. 23993680 10.1016/j.clinph.2013.06.187 PMC3954153

[90] K. Mills , S. Boniface , M. Schubert , Electroencephalogr. Clin. Neurophysiol./Evoked Potentials Sect. 1992, 85, 17. 10.1016/0168-5597(92)90096-t 1371739

[91] J. B. Silveira , R. F. Damiano , E. Abelama Neto , R. N. d. S. M. Gomes , I. Klein , L. Borrione , P. Sudbrack , A. F. Gentil , E. Shephard , A. R. Brunoni , Double cone coil repetitive transcranial magnetic stimulation for severe obsessive‐compulsive disorder after reversible cerebral vasoconstriction syndrome with intracerebral hemorrhage: a case report, SciELO, Brasil: 2022. 10.47626/1516-4446-2022-2556 PMC9561833 36423361

[92] S. Vanneste , D. De Ridder , Brain Stimul. 2013, 6, 155. 22658239 10.1016/j.brs.2012.03.019

[93] S. Vanneste , M. Plazier , P. Van de Heyning , D. De Ridder , J. Neurol., Neurosurg. Psychiat. 2011, 82, 1160. 21429905 10.1136/jnnp.2010.213959

[94] N. Branston , P. Tofts , Phys. Med. Biol. 1991, 36, 161.

[95] P. Tofts , Phys. Med. Biol. 1990, 35, 1119. 2217537 10.1088/0031-9155/35/8/008

[96] C. Ren , P. P. Tarjan , D. B. Popovic , IEEE Trans. Biomed. Eng. 1995, 42, 918. 7558066 10.1109/10.412658

[97] K. P. Zimmermann , R. K. Simpson , Electroencephalogr. Clin. Neurophysiol./Electromyogr. Motor Control 1996, 101, 145. 10.1016/0924-980x(95)00227-c 8647019

[98] Y. Roth , A. Zangen , M. Hallett , J. Clin. Neurophysiol. 2002, 19, 361. 12436090 10.1097/00004691-200208000-00008

[99] A. Zangen , Y. Roth , B. Voller , M. Hallett , Clin. Neurophysiol. 2005, 116, 775. 15792886 10.1016/j.clinph.2004.11.008

[100] Y. Roth , A. Amir , Y. Levkovitz , A. Zangen , J. Clin. Neurophysiol. 2007, 24, 31. 17277575 10.1097/WNP.0b013e31802fa393

[101] E. V. Harel , A. Zangen , Y. Roth , I. Reti , Y. Braw , Y. Levkovitz , World J. Biol. Psychiat. 2011, 12, 119. 10.3109/15622975.2010.510893 20854181

[102] E. V. Harel , L. Rabany , L. Deutsch , Y. Bloch , A. Zangen , Y. Levkovitz , World J. Biol. Psychiat. 2014, 15, 298. 10.3109/15622975.2011.639802 22313023

[103] E. Onesti , M. Gabriele , C. Cambieri , M. Ceccanti , R. Raccah , G. Di Stefano , A. Biasiotta , A. Truini , A. Zangen , M. Inghilleri , Eur. J. Pain 2013, 17, 1347. 23629867 10.1002/j.1532-2149.2013.00320.x

[104] Y. Meng , R. L. Hadimani , L. J. Crowther , Z. Xu , J. Qu , D. Jiles , J. Appl. Phys. 2015, 117.

[105] P. Rastogi , E. G. Lee , R. L. Hadimani , D. C. Jiles , IEEE Magn. Lett. 2019, 10, 3102205.

[106] J. Ruohonen , P. Ravazzani , F. Grandori , R. J. Ilmoniemi , IEEE Trans. Biomed. Eng. 1999, 46, 646. 10356871 10.1109/10.764941

[107] B. H. Han , I. K. Chun , S. C. Lee , S. Y. Lee , IEEE Trans. Biomed. Eng. 2004, 51, 812. 15132507 10.1109/TBME.2004.824123

[108] S. Yang , G. Xu , L. Wang , Y. Geng , H. Yu , Q. Yang , IEEE Trans. Appl. Supercond. 2010, 20, 829.

[109] X. Wei , Y. Li , M. Lu , J. Wang , G. Yi , Int. J. Environ. Res. Public Health 2017, 14, 1388. 29135963 10.3390/ijerph14111388 PMC5708027

[110] W. Rushton , J. Physiol. 1927, 63, 357. 16993895 10.1113/jphysiol.1927.sp002409 PMC1514939

[111] J. B. Ranck, Jr , Brain Res. 1975, 98, 417. 1102064 10.1016/0006-8993(75)90364-9

[112] K. Sakai , Y. Ugawa , Y. Terao , R. Hanajima , T. Furubayashi , I. Kanazawa , Exp. Brain Res. 1997, 113, 24. 9028772 10.1007/BF02454139

[113] A. A. d'Alfonso , J. van Honk , E. Hermans , A. Postma , E. H. de Haan , Neurosci. Lett. 2000, 280, 195. 10675794 10.1016/s0304-3940(00)00781-3

[114] S. W. Lee , S. I. Fried , IEEE Trans. Neural Syst. Rehabil. Eng. 2016, 25, 1375. 27893396 10.1109/TNSRE.2016.2631446 PMC5498237

[115] S. W. Lee , S. I. Fried , IEEE Trans. Neural Syst. Rehabil. Eng. 2017, 25, 1375. 27893396 10.1109/TNSRE.2016.2631446 PMC5498237

[116] S. B. Ryu , A. C. Paulk , J. C. Yang , M. Ganji , S. A. Dayeh , S. S. Cash , S. I. Fried , S. W. Lee , J. Neural Eng. 2020, 17, 056036. 32998116 10.1088/1741-2552/abbd22 PMC8923513

[117] S. W. Lee , S. I. Fried , presented at 2013 6th International IEEE/EMBS Conference on Neural Engineering (NER) , 2013.

[118] M. Zaeimbashi , Z. Wang , S. W. Lee , S. Cash , S. Fried , N. Sun , presented at 2018 40th Annual International Conference of the IEEE Engineering in Medicine and Biology Society (EMBC) , 2018. 10.1109/EMBC.2018.8512729 30440849

[119] A. Khalifa , M. Zaeimbashi , T. X. Zhou , S. M. Abrishami , N. Sun , S. Park , T. Šumarac , J. Qu , I. Zohar , A. Yacoby , S. Cash , N. X. Sun , Microsyst. Nanoeng. 2021, 7, 91. 34786205 10.1038/s41378-021-00320-8 PMC8589949

[120] H. Ye , V. Hall , J. Hendee , Front. Comput. Neurosci. 2023, 17, 1105505. 36817316 10.3389/fncom.2023.1105505 PMC9932264

[121] S. Minusa , H. Osanai , T. Tateno , IEEE Trans. Biomed. Eng. 2017, 65, 1301. 28880154 10.1109/TBME.2017.2748136

[122] R. Saha , S. Faramarzi , R. P. Bloom , O. J. Benally , K. Wu , A. Di Girolamo , D. Tonini , S. A. Keirstead , W. C. Low , T. I. Netoff , J. Neural Eng. 2022, 19, 016018. 10.1088/1741-2552/ac4baf 35030549

[123] L. Golestanirad , J. T. Gale , N. F. Manzoor , H.‐J. Park , L. Glait , F. Haer , J. A. Kaltenbach , G. Bonmassar , Front. Physiol. 2018, 9, 724. 30140230 10.3389/fphys.2018.00724 PMC6094965

[124] G. Bonmassar , P. Serano , Front. Hum. Neurosci. 2020, 14, 53. 32231526 10.3389/fnhum.2020.00053 PMC7082860

[125] T. Kim , H. Kadji , A. J. Whalen , A. Ashourvan , E. Freeman , S. I. Fried , S. Tadigadapa , S. J. Schiff , J. Neural Eng. 2022, 19, 056029. 10.1088/1741-2552/ac9339 PMC9855718 36126646

[126] M. Rizou , T. Prodromakis , presented at 2016 IEEE Biomedical Circuits and Systems Conference (BioCAS) , 2016.

[127] M.‐E. Rizou , T. Prodromakis , Biomed. Phys. Eng. Express 2018, 4, 025016.

[128] G. Bonmassar , in 2017 Int. Conf. on Electromagnetics in Advanced Applications (ICEAA) IEEE , 2017, pp. 1875–1878.

[129] G. Bonmassar , L. Golestanirad , J. Deng , MRS Adv. 2018, 3, 1635. 31105970 10.1557/adv.2018.155 PMC6519713

[130] R. Bernardo , A. Rodrigues , M. P. S. Dos Santos , P. Carneiro , A. Lopes , J. S. Amaral , V. S. Amaral , R. Morais , Med. Eng. Phys. 2019, 73, 77. 31477429 10.1016/j.medengphy.2019.07.015

[131] L. Tian , L. Song , Y. Zheng , J. Wang , H. Chen , IEEE Trans. Magn. 2020, 56, 4001008.

[132] H. Jeong , A. Cho , I. Ay , G. Bonmassar , Front. Physiol. 2022, 13, 938101. 36277182 10.3389/fphys.2022.938101 PMC9585240

[133] H. Jeong , J. Deng , G. Bonmassar , Journal of Vacuum Science & Technology B 2021, 39. 10.1116/6.0001281 PMC8516478 34692236

[134] H.‐J. Park , J. H. Seol , J. Ku , S. Kim , IEEE Trans. Biomed. Eng. 2016, 63, 158. 26468905 10.1109/TBME.2015.2490244

[135] C. Ge , F. Walton , W. Xu , H. Heidari , presented at 2022 2022.

[136] E. C. Szoka , J. C. Werth , S. Lee , J.‐I. Lee , A. J. Cortese , T. A. Cleland , S. Fried , A. Molnar , presented at 2021 10th International IEEE/EMBS Conference on Neural Engineering (NER) , 2021.

[137] S. W. Lee , S. I. Fried , Philos. Trans. R. Soc. A 2022, 380, 20210019. 10.1098/rsta.2021.0019 PMC11493182 35658677

[138] K. Thyagarajan , R. A. Lujan , Q. Wang , J. Lu , S. Kor , B. Kakimoto , N. Chang , J. A. Bert , APL Mater. 2021, 9. 10.1063/5.0023486 PMC7808331 33520428

[139] A. J. Whalen , S. I. Fried , J. Neural Eng. 046017, 2023. 10.1088/1741-2552/ace79a PMC10467159 37451256

[140] G. Bonmassar , J. Gale , W. Vanduffel , Int. J. Bioelectromagn. 2014, 16.

[141] M. E. Rizou , T. Prodromakis , Biomed. Phys. Eng. Express 2018, 4, 25016.

[142] S. Minusa , S. Muramatsu , H. Osanai , T. Tateno , J. Neural Eng. 2019, 16, 066014. 31642445 10.1088/1741-2552/ab3187

[143] Y. Zheng , Q. Liu , Y. Zhao , Y. Qi , L. Dong , Methods 2024, 229, 49. 38880432 10.1016/j.ymeth.2024.06.004

[144] S. W. Lee , IEEE Trans. Biomed. Eng. 2020, 68, 2164. 10.1109/TBME.2020.3033491 PMC8262115 33095707

[145] V. Raghuram , A. D. Datye , S. I. Fried , B. P. Timko , bioRxiv 2021, 2021.12. 07.471184.

[146] S. Sugai , H. Higuchi , J. Nishikawa , K. Satoh , S. Murakami , T. Tateno , IEEJ Trans. Electr. Electron. Eng. 2020, 15, 1672.

[147] G. Bonmassar , S. W. Lee , D. K. Freeman , M. Polasek , S. I. Fried , J. T. Gale , Nat. Commun. 2012, 3, 921 22735449 10.1038/ncomms1914 PMC3621430

[148] S. W. Lee , S. I. Fried , in 2014 36th Annual Int. Conf. of the IEEE Engineering in Medicine and Biology Society IEEE , 2014, pp. 6125–6128. 10.1109/EMBC.2014.6945027 PMC4465444 25571395

[149] M. P. Calatayud , B. Sanz , V. Raffa , C. Riggio , M. R. Ibarra , G. F. Goya , Biomaterials 2014, 35, 6389. 24816288 10.1016/j.biomaterials.2014.04.009

[150] S. A. Blank‐Shim , S. P. Schwaminger , M. Borkowska‐Panek , P. Anand , P. Yamin , P. Fraga‐García , K. Fink , W. Wenzel , S. Berensmeier , Sci. Rep. 2017, 7, 14047. 29070786 10.1038/s41598-017-13928-6 PMC5656586

[151] R. Weissleder , K. Kelly , E. Y. Sun , T. Shtatland , L. Josephson , Nat. Biotechnol. 2005, 23, 1418. 16244656 10.1038/nbt1159

[152] J. Dobson , Nat. Nanotechnol. 2008, 3, 139. 18654485 10.1038/nnano.2008.39

[153] D. Ortega , Q. A. Pankhurst , Nanoscience 2013, 1, e88.

[154] A. E. Deatsch , B. A. Evans , J. Magn. Magn. Mater. 2014, 354, 163.

[155] K. Mahmoudi , A. Bouras , D. Bozec , R. Ivkov , C. Hadjipanayis , Int. J. Hypertherm. 2018, 34, 1316. 10.1080/02656736.2018.1430867 PMC6078833 29353516

[156] R. Chen , G. Romero , M. G. Christiansen , A. Mohr , P. Anikeeva , Science 2015, 347, 1477. 25765068 10.1126/science.1261821

[157] R. Munshi , S. M. Qadri , Q. Zhang , I. Castellanos Rubio , P. Del Pino , A. Pralle , eLife 2017, 6. 10.7554/eLife.27069 PMC5779110 28826470

[158] D. Rosenfeld , H. Field , Y. J. Kim , K. K. L. Pang , K. Nagao , F. Koehler , P. Anikeeva , Adv. Funct. Mater. 2022, 32, 0224558.

[159] D. Rosenfeld , A. W. Senko , J. Moon , I. Yick , G. Varnavides , D. Gregureć , F. Koehler , P.‐H. Chiang , M. G. Christiansen , L. Y. Maeng , A. S. Widge , P. Anikeeva , Sci. Adv. 2020, 6, eaaz3734. 32300655 10.1126/sciadv.aaz3734 PMC7148104

[160] P. Chandrasekharan , Z. W. Tay , D. Hensley , X. Y. Zhou , B. K. Fung , C. Colson , Y. Lu , B. D. Fellows , Q. Huynh , C. Saayujya , E. Yu , R. Orendorff , B. Zheng , P. Goodwill , C. Rinaldi , S. Conolly , Theranostics 2020, 10, 2965. 32194849 10.7150/thno.40858 PMC7053197

[161] S. Min , M. J. Ko , H. J. Jung , W. Kim , S. B. Han , Y. Kim , G. Bae , S. Lee , R. Thangam , H. Choi , Adv. Mater. 2021, 33, 2008353. 10.1002/adma.202008353 33527502

[162] S. Min , Y. S. Jeon , H. J. Jung , C. Khatua , N. Li , G. Bae , H. Choi , H. Hong , J. E. Shin , M. J. Ko , Adv. Mater. 2020, 32, 2004300. 10.1002/adma.202004300 32820574

[163] L. S. Ganapathe , M. A. Mohamed , R. M. Yunus , D. D. Berhanuddin , Magnetochemistry 2020, 6, 68.

[164] M. D. Nguyen , H.‐V. Tran , S. Xu , T. R. Lee , Appl. Sci. 2021, 11, 11301. 35844268 10.3390/app112311301 PMC9285867

[165] K. N. Koo , A. F. Ismail , M. H. D. Othman , N. Bidin , M. A. Rahman , Malaysian J. Fundam. Appl. Sci. 2019, 15, 23.

[166] N. Tran , T. J. Webster , J. Mater. Chem. 2010, 20, 8760.

[167] M. Srivastava , S. Alla , S. S. Meena , N. Gupta , R. Mandal , N. Prasad , New J. Chem. 2018, 42, 7144.

[168] P. Saha , R. Rakshit , K. Mandal , J. Magn. Magn. Mater. 2019, 475, 130.

[169] J. t. Jang , H. Nah , J. H. Lee , S. H. Moon , M. G. Kim , J. Cheon , Angew. Chem., Int. Ed. 2009, 48, 1234. 10.1002/anie.200805149 19137514

[170] K. Chatterjee , S. Sarkar , K. Jagajjanani Rao , S. Paria , Adv. Colloid Interface Sci. 2014, 209, 8. 24491963 10.1016/j.cis.2013.12.008

[171] L.‐M. Lacroix , N. Frey Huls , D. Ho , X. Sun , K. Cheng , S. Sun , Nano Lett. 2011, 11, 1641. 21417366 10.1021/nl200110t

[172] L. D. Marks , L. Peng , J. Phys.: Condens. Matter 2016, 28, 053001. 26792459 10.1088/0953-8984/28/5/053001

[173] K. L. Kelly , E. Coronado , L. L. Zhao , G. C. Schatz , J. Phys. Chem. B 2003, 107, 668.

[174] Z. Nemati , S. Salili , J. Alonso , A. Ataie , R. Das , M. Phan , H. Srikanth , J. Alloys Compd. 2017, 714, 709.

[175] R. G. D. Andrade , S. R. S. Veloso , E. M. S. Castanheira , Int. J. Mol. Sci. 2020, 21, 2455. 32244817 10.3390/ijms21072455 PMC7178053

[176] D. Lisjak , A. Mertelj , Prog. Mater. Sci. 2018, 95, 286.

[177] J. Borch , T. Hamann , 2009, 10.1515/BC.2009.091.

[178] G. Zou , K. Xiong , C. Jiang , H. Li , Y. Wang , S. Zhang , Y. Qian , Nanotechnology 2005, 16, 1584.

[179] M. Hilse , J. Herfort , B. Jenichen , A. Trampert , M. Hanke , P. Schaaf , L. Geelhaar , H. Riechert , Nano Lett. 2013, 13, 6203. 24274677 10.1021/nl4035994

[180] T. Zhang , H. Wang , X. Guo , S. Shao , L. Ding , A. Han , L. Wang , J. Liu , Appl. Catal., B 2022, 304, 120925.

[181] L. Signorelli , S.‐A. Hescham , A. Pralle , D. Gregurec , iScience 2022, 25, 105401. 36388996 10.1016/j.isci.2022.105401 PMC9641224

[182] S. Bevan , T. Quallo , D. A. Andersson , Mammalian Transient Receptor Potential (TRP) Cation Channels 2014, I, 207.

[183] M. Tominaga , T. Tominaga , Pflügers Archiv 2005, 451, 143. 15971082 10.1007/s00424-005-1457-8

[184] T. D. Plant , R. Strotmann , Handbk Exp. Pharmacol. 2007, 189. 10.1007/978-3-540-34891-7_11 17217058

[185] M.‐K. Chung , H. Lee , M. J. Caterina , J. Biol. Chem. 2003, 278, 32037. 12783886 10.1074/jbc.M303251200

[186] H. Todaka , J. Taniguchi , J.‐i. Satoh , A. Mizuno , M. Suzuki , J. Biol. Chem. 2004, 279, 35133. 15187078 10.1074/jbc.M406260200

[187] E. R. Schneider , E. O. Anderson , E. O. Gracheva , S. N. Bagriantsev , Curr. Top. Membr. 2014, 74, 113. 25366235 10.1016/B978-0-12-800181-3.00005-1 PMC4794111

[188] F. N. Hamada , M. Rosenzweig , K. Kang , S. R. Pulver , A. Ghezzi , T. J. Jegla , P. A. Garrity , Nature 2008, 454, 217. 18548007 10.1038/nature07001 PMC2730888

[189] M. Rosenzweig , K. M. Brennan , T. D. Tayler , P. O. Phelps , A. Patapoutian , P. A. Garrity , Genes Dev. 2005, 19, 419. 15681611 10.1101/gad.1278205 PMC548941

[190] Y. Yu , C. Payne , N. Marina , A. Korsak , P. Southern , A. García‐Prieto , I. N. Christie , R. R. Baker , E. M. Fisher , J. A. Wells , Adv. Sci. 2022, 9, 2104194. 10.1002/advs.202104194 PMC8867145 34927381

[191] M. Szczot , A. R. Nickolls , R. M. Lam , A. T. Chesler , Annu. Rev. Biochem. 2021, 90, 507. 34153212 10.1146/annurev-biochem-081720-023244 PMC8794004

[192] Y.‐C. Lin , Y. R. Guo , A. Miyagi , J. Levring , R. MacKinnon , S. Scheuring , Nature 2019, 573, 230. 31435018 10.1038/s41586-019-1499-2 PMC7258172

[193] P. A. Gottlieb , C. Bae , F. Sachs , Channels 2012, 6, 282. 22790451 10.4161/chan.21064 PMC3508907

[194] Y. Jiang , X. Yang , J. Jiang , B. Xiao , Trends Biochem. Sci. (Amsterdam. Regular ed.) 2021, 46, 472. 10.1016/j.tibs.2021.01.008 33610426

[195] Y. R. Guo , R. MacKinnon , eLife 2017, 6. 10.7554/eLife.33660 PMC5788504 29231809

[196] G. Calixto , J. Bernegossi , B. Fonseca‐Santos , M. Chorilli , Int. J. Nanomed. 2014, 9, 3719. 10.2147/IJN.S61670 PMC4134022 25143724

[197] A. Garcia‐Elias , S. Mrkonjić , C. Jung , C. Pardo‐Pastor , R. Vicente , M. A. Valverde , Handbk Exp. Pharmacol. 2014, 222, 293. 10.1007/978-3-642-54215-2_12 24756711

[198] G. Du , L. Li , X. Zhang , J. Liu , J. Hao , J. Zhu , H. Wu , W. Chen , Q. Zhang , Exp. Biol. Med. (Maywood, N.J.) 2020, 245, 180. 10.1177/1535370219892601 PMC7045327 31791130

[199] J. M. Kanczler , H. S. Sura , J. Magnay , D. Green , R. O. Oreffo , J. P. Dobson , A. J. El Haj , Tissue Eng., Part A 2010, 16, 3241. 20504072 10.1089/ten.TEA.2009.0638

[200] F. Maingret , M. Fosset , F. Lesage , M. Lazdunski , E. Honoré , J. Biol. Chem. 1999, 274, 1381. 9880510 10.1074/jbc.274.3.1381

[201] S. G. Brohawn , E. B. Campbell , R. MacKinnon , Nature 2014, 516, 126. 25471887 10.1038/nature14013 PMC4682367

[202] J. McCarthy , X. Gong , D. Nahirney , M. Duszyk , M. Radomski , Int. J. Nanomed. 2011, 1343. 10.2147/IJN.S21145 PMC3133525 21760729

[203] P. Huang , H. C. Chan , W. K. Zhang , D. Wang , Y. Duan , M. M. T. Loy , Nat. Cell Biol. 2010, 12, 507. 20400957 10.1038/ncb2053

[204] Y. Yu , C. Payne , N. Marina , A. Korsak , P. Southern , A. García‐Prieto , I. N. Christie , R. R. Baker , E. M. C. Fisher , J. A. Wells , T. L. Kalber , Q. A. Pankhurst , A. V. Gourine , M. F. Lythgoe , Adv. Sci. 2022, 9, e2104194. 10.1002/advs.202104194 PMC8867145 34927381

[205] C.‐L. Su , C.‐C. Cheng , P.‐H. Yen , J.‐X. Huang , Y.‐J. Ting , P.‐H. Chiang , Commun. Biol. 2022, 5, 1166. 36323817 10.1038/s42003-022-04124-y PMC9630493

[206] J.‐H. Lee , J.‐t. Jang , J.‐s. Choi , S. H. Moon , S.‐h. Noh , J.‐w. Kim , J.‐G. Kim , I.‐S. Kim , K. I. Park , J. Cheon , Nat. Nanotechnol. 2011, 6, 418. 21706024 10.1038/nnano.2011.95

[207] D. Yoo , J.‐H. Lee , T.‐H. Shin , J. Cheon , Acc. Chem. Res. 2011, 44, 863. 21823593 10.1021/ar200085c

[208] L. Chen , C. Chen , P. Wang , T. Song , J. Nanomater. 2017, 2017, 1564634.

[209] J.‐u. Lee , W. Shin , Y. Lim , J. Kim , W. R. Kim , H. Kim , J.‐H. Lee , J. Cheon , Nat. Mater. 2021, 20, 1029. 33510447 10.1038/s41563-020-00896-y

[210] Brainstorm Project , https://www.brainstorm‐project.eu/https://cordis.europa.eu/project/id/101099355 (accessed: December 2023).

[211] S. Wu , H. Li , D. Wang , L. Zhao , X. Qiao , X. Zhang , W. Liu , C. Wang , J. Zhou , Nano Today 2021, 39, 101187.

[212] H. Kim , J. Kim , J. Kim , S. Oh , K. Choi , J. Yoon , Sci. Rep. 2023, 13, 4988. 36973390 10.1038/s41598-023-31979-w PMC10042827

[213] F. Khan , N. S. Khattak , U. S. Khan , A. Rahman , J. Chem. Soc. Pak. 2011, 33, 793.

[214] A.‐G. Niculescu , C. Chircov , A. M. Grumezescu , Methods 2022, 199, 16. 33915292 10.1016/j.ymeth.2021.04.018

[215] J. Carrey , B. Mehdaoui , M. Respaud , J. Appl. Phys. 2011, 109, 083921.

[216] S. Yoffe , T. Leshuk , P. Everett , F. Gu , Curr. Pharm. Des. 2013, 19, 493. 22934920

[217] G. Indech , L. Geri , C. Mordechai , Y. Ben Moshe , Y. Mastai , O. Shefi , A. Sharoni , J. Mater. Chem. B: Mater. Biol. Med. 2023, 11, 7094. 37016795 10.1039/d2tb02456j

[218] F. Ozel , H. Kockar , J. Magn. Magn. Mater. 2015, 373, 213.

[219] V. Sundaresan , J. U. Menon , M. Rahimi , K. T. Nguyen , A. S. Wadajkar , Int. J. Pharm. 2014, 466, 1. 24607216 10.1016/j.ijpharm.2014.03.016 PMC4642438

[220] D. Gregurec , A. W. Senko , A. Chuvilin , P. D. Reddy , A. Sankararaman , D. Rosenfeld , P.‐H. Chiang , F. Garcia , I. Tafel , G. Varnavides , E. Ciocan , P. Anikeeva , ACS Nano 2020, 14, 8036. 32559057 10.1021/acsnano.0c00562 PMC8592276

[221] C.‐J. Jia , L.‐D. Sun , F. Luo , X.‐D. Han , L. J. Heyderman , Z.‐G. Yan , C.‐H. Yan , K. Zheng , Z. Zhang , M. Takano , N. Hayashi , M. Eltschka , M. Kläui , U. Rüdiger , T. Kasama , L. Cervera‐Gontard , R. E. Dunin‐Borkowski , G. Tzvetkov , J. r. Raabe , J. Am. Chem. Soc. 2008, 130, 16968. 19053430 10.1021/ja805152t

[222] Z. Wang , Microelectron. Eng. 2019, 210, 35.

[223] J. P. Gambino , S. A. Adderly , J. U. Knickerbocker , Microelectron. Eng. 2015, 135, 73.

[224] O. Seong Joon , K. Chunho , D. F. Baldwin , IEEE Trans. Advan. Packaging 2003, 26, 302.

[225] E. M. Chow , V. Chandrasekaran , A. Partridge , T. Nishida , M. Sheplak , C. F. Quate , T. W. Kenny , J. Microelectromech. Syst. 2002, 11, 631.

[226] P. Dixit , J. Miao , R. Preisser , Electrochem. Solid‐State Lett. 2006, 9, G305.

[227] G. Parès , S. Minoret , J. F. Lugand , S. Huet , V. Lapras , R. Anciant , D. Henry , N. Sillon , B. Dunne , in *2009 11th Electronics Packaging Technology Conf*. 2009, pp. 772–777.

[228] T. Xu , J. Sun , H. Wu , H. Li , H. Li , Z. Tao , IEEE Electron Device Lett. 2019, 40, 1816.

[229] T. Pan , A. Baldi , E. Davies‐Venn , R. F. Drayton , B. Ziaie , J. Micromech. Microeng. 2005, 15, 849.

[230] M. Wang , I. Batarseh , K. D. T. Ngo , H. Xie , in *2007 IEEE Power Electronics Specialists Conf*. 2007, p. 1612.

[231] M. Wang , K. D. T. Ngo , H. Xie in 2008 34th Annual Conf. of IEEE Industrial Electronics 2008, p. 2672.

[232] M. J. K. Klein , T. Ono , M. Esashi , J. G. Korvink , J. Micromech. Microeng. 2008, 18, 075002.

[233] C. Zhuo , C. Baixin , presented at 2017 China Semiconductor Technology International Conference (CSTIC) 12 March 2017 2017.

[234] J. Li , K. D. T. Ngo , G. Q. Lu , H. Xie , presented at 2012 7th International Conference on Integrated Power Electronics Systems (CIPS) , 6–8 March 2012 2012.

[235] J. Li , V. F. G. Tseng , Z. Xiao , H. Xie , IEEE Trans. Power Electron. 2017, 32, 3858.

[236] S. W. Lee , S. I. Fried , in 2015 7th Int. IEEE/EMBS Conf. on Neural Engineering (NER) 2015, p. 268.

[237] J. M. Bustillo , R. T. Howe , R. S. Muller , Proc. IEEE 1998, 86, 1552.

[238] D.‐M. Fang , Y. Zhou , W. Ding , X.‐N. Wang , X.‐L. Zhao , Surface Micromachined Three‐Dimensional Solenoid‐Type Inductor, Elsevier, Oxford 2006.

[239] Y. Jun‐Bo , C. Yun‐Seok , K. Byeong‐Il , E. Yunseong , Y. Euisik , IEEE Electron Device Lett. 2002, 23, 591.

[240] K. Yong‐Jun , M. G. Allen , IEEE Tran. Comp., Packaging, Manuf. Technol., Part C 1998, 21, 26.

[241] M. Wang , J. Li , K. D. T. Ngo , H. Xie , IEEE Trans. Power Electron. 2011, 26, 1310.

[242] Y. Jun‐Bo , K. Bon‐Kee , H. Chul‐Hi , Y. Euisik , K. Choong‐Ki , IEEE Electron Device Lett. 1999, 20, 487.

[243] Y. Shim , Z. Wu , M. Rais‐Zadeh , J. Microelectromech. Syst. 2012, 21, 867.

[244] P. Jin‐Woo , F. Cros , M. G. Allen , in Technical Digest. MEMS 2002 IEEE Int. Conf. Fifteenth IEEE Int. Conf. on Micro Electro Mechanical Systems (Cat. No.02CH37266) 2002, pp. 380–383.

[245] M. Gel , S. Takeuchi , I. Shimoyama , Sens. Actuators, A 2002, 97, 702.

[246] D. R. Hines , N. P. Siwak , L. A. Mosher , R. Ghodssi (Eds: R. Ghodssi , P. Lin ), Springer US, Boston, MA 2011.

[247] R. Ballarini , H. Kahn , A. H. Heuer , M. P. De Boer , M. T. Dugger , in (Eds: I. Milne , R. O. Ritchie , B. Karihaloo ), Pergamon, Oxford 2003.

[248] R. P. Ribas , J. Lescot , J. L. Leclercq , J. M. Karam , F. Ndagijimana , IEEE Trans. Microw. Theory Techn. 2000, 48, 1326.

[249] J. Hongrui , W. Ye , J. L. A. Yeh , N. C. Tien , IEEE Trans. Microwave Theory Techn. 2000, 48, 2415.

[250] C. H. Ahn , M. G. Allen , IEEE Trans. Ind. Electron. 1998, 45, 866.

[251] T. S. D. Cheung , J. R. Long , IEEE J. Solid‐State Circuits 2006, 41, 1183.

[252] I. Papagiannopoulos , V. Chatziathanasiou , A. Hatzopoulos , M. Kałuża , B. Wie¸cek , G. De Mey , Infrared Phys. Technol. 2013, 56, 80.

[253] H. Zhang , P. Machura , K. Kails , H. Chen , M. Mueller , Supercond. Sci. Technol. 2020, 33, 084008.

[254] P. M. Aguiar , J.‐F. Jacquinot , D. Sakellariou , J. Magn. Reson. 2009, 200, 6. 19541518 10.1016/j.jmr.2009.05.010

[255] D.‐H. Weon , J.‐H. Jeon , S. Mohammadi , J. Vac. Sci. Technol. B: Microelectron. Nanometer Struct. Process., Meas., Phenom. 2007, 25, 264.

[256] A. C. Siegel , D. A. Bruzewicz , D. B. Weibel , G. M. Whitesides , Adv. Mater. 2007, 19, 727.

[257] A. Moazenzadeh , N. Spengler , U. Wallrabe , in 2013 IEEE 26th International Conference on Micro Electro Mechanical Systems (MEMS) , 2013, 287.

[258] S. H. Lee , R. D. Lorenz , in 2013 Twenty‐Eighth Annual IEEE Appl. Power Electronics Conf. and Exposition (APEC) 2013, p. 1783.

[259] X. Liu , A. J. Whalen , S. B. Ryu , S. W. Lee , S. I. Fried , K. Kim , C. Cai , M. Lauritzen , N. Bertram , B. Chang , T. Yu , A. Han , Biosens. Bioelectron. 2023, 227, 115143. 36805270 10.1016/j.bios.2023.115143

[260] S. Minusa , H. Osanai , T. Tateno , IEEE Trans. Biomed. Eng. 2018, 65, 1301. 28880154 10.1109/TBME.2017.2748136

[261] W. Jiang , R. Isenhart , N. Kistler , Z. Lu , H. Xu , D. J. Lee , C. Y. Liu , D. Song , in 2021 43rd Annual Int. Conf. of the IEEE Engineering in Medicine & Biology Society (EMBC) 2021, p. 6318. 10.1109/EMBC46164.2021.9630986 34892558

[262] M. Kabar , L. Lopez‐Chau , Brain Stimul.: Basic, Transl., Clin. Res. Neuromodul. 2021, 14, 1603.

[263] M. Javaid , A. Haleem , R. P. Singh , R. Suman , S. Rab , Adv. Ind. Eng. Polym. Res. 2021, 4, 312.

[264] G. N. Levy , R. Schindel , J. P. Kruth , CIRP Ann. 2003, 52, 589.

[265] K. Wang , Q. Ma , C.‐X. Qu , H.‐T. Zhou , M. Cao , S.‐D. Wang , AUTEX Res. J. 2023, 23, 350.

[266] D. Qin , Y. Xia , G. M. Whitesides , Nat. Protoc. 2010, 5, 491. 20203666 10.1038/nprot.2009.234

[267] R. A. Lawson , A. P. G. Robinson , in (Eds: A. Robinson , R. Lawson ), Elsevier, Amsterdam, Netherlands 2016.

[268] A. Tritchkov , R. Jonckheere , L. Van den hove , J. Vac. Sci. Technol. B: Microelectron. Nanometer Struct. Process., Meas., Phenom. 1995, 13, 2986.

[269] S. Ghosh , C. P. Pradeep , S. K. Sharma , P. G. Reddy , S. P. Pal , K. E. Gonsalves , RSC Adv. 2016, 6, 74462.

[270] E. H. Waller , G. v. Freymann , Nanophotonics 2018, 7, 1259.

[271] X. Zheng , K. Cheng , X. Zhou , J. Lin , X. Jing , AIP Adv. 2017, 7.

[272] F. Sima , K. Sugioka , R. M. Vázquez , R. Osellame , L. Kelemen , P. Ormos , Nanophotonics 2018, 7, 613.

[273] M. Rothschild , Mater. Today 2005, 8, 18.

[274] H. Hassanin , G. Sheikholeslami , P. Sareh , R. B. Ishaq , Adv. Eng. Mater. 2021, 23, 2100422.

[275] Y. Matsumoto , M. Setomoto , D. Noda , T. Hattori , Microsyst. Technol. 2008, 14, 1373.

[276] C. W. Ha , P. Prabhakaran , Y. Son , 3D Print. Addit. Manuf. 2019, 6, 165.

[277] K. Stokes , K. Clark , D. Odetade , M. Hardy , P. G. Oppenheimer , Discov. Nano 2023, 18, 153. 38082047 10.1186/s11671-023-03938-x PMC10713959

[278] B. J. Barros , J. P. S. Cunha , Front. Neurosci. 2024, 18. 10.3389/fnins.2024.1382341 PMC11102054 38765670

[279] M. E. Rizou , T. Prodromakis , in 2016 IEEE Biomedical Circuits and Systems Conf. (BioCAS) 2016, 34.

[280] D. J. Oathes , N. L. Balderston , K. P. Kording , J. A. DeLuisi , G. M. Perez , J. D. Medaglia , Y. Fan , R. J. Duprat , T. D. Satterthwaite , Y. I. Sheline , K. A. Linn , WIREs Cogn. Sci. 2021, 12, e1553. 10.1002/wcs.1553 PMC8521438 33470055

[281] M. B. Kennedy , Cold Spring Harb. Perspect. Biol. 2013, 8, a016824. 24379319 10.1101/cshperspect.a016824 PMC4743082

[282] J. G. Howland , Y. T. Wang , in (Eds: W. S. Sossin , J.‐C. Lacaille , V. F. Castellucci , S. Belleville ), Elsevier, Amsterdam, Netherlands 2008.

[283] H.‐J. Park , H. Kang , J. Jo , E. Chung , S. Kim , Sci. Rep. 2018, 8, 13423. 30194395 10.1038/s41598-018-31536-w PMC6128857

[284] M. Huth , F. Porrati , O. V. Dobrovolskiy , Microelectron. Eng. 2018, 185–186, 9.

[285] R. Winkler , J. D. Fowlkes , P. D. Rack , H. Plank , J. Appl. Phys. 2019, 125.

[286] P. Orús , F. Sigloch , S. Sangiao , J. M. De Teresa , Nanomaterials 2022, 12, 1367. 35458074 10.3390/nano12081367 PMC9029853

[287] A. Fernández‐Pacheco , L. Skoric , J. M. De Teresa , J. Pablo‐Navarro , M. Huth , O. V. Dobrovolskiy , Materials 2020, 13, 3774. 32859076 10.3390/ma13173774 PMC7503546

[288] M. Toth , C. Lobo , V. Friedli , A. Szkudlarek , I. Utke , Beilstein J. Nanotechnol. 2015, 6, 1518. 26425405 10.3762/bjnano.6.157 PMC4578401

[289] W. F. van Dorp , C. W. Hagen , J. Appl. Phys. 2008, 104.

[290] R. Córdoba , N. Sharma , S. Kölling , P. M. Koenraad , B. Koopmans , Nanotechnology 2016, 27, 355301. 27454835 10.1088/0957-4484/27/35/355301

[291] M. Jaafar , J. Pablo‐Navarro , E. Berganza , P. Ares , C. Magén , A. Masseboeuf , C. Gatel , E. Snoeck , J. Gómez‐Herrero , J. M. de Teresa , A. Asenjo , Nanoscale 2020, 12, 10090. 32348391 10.1039/d0nr00322k

[292] H. Mattiat , N. Rossi , B. Gross , J. Pablo‐Navarro , C. Magén , R. Badea , J. Berezovsky , J. M. De Teresa , M. Poggio , Phys. Rev. Appl. 2020, 13, 044043.

[293] I. Utke , J. Michler , R. Winkler , H. Plank , Micromachines 2020, 11, 397. 32290292 10.3390/mi11040397 PMC7231341

[294] B. J. Nelson , L. Dong , in (Ed: B. Bhushan ), Springer, Berlin, Heidelberg 2010.

[295] R. Córdoba , A. Ibarra , D. Mailly , J. M. De Teresa , Nano Lett. 2018, 18, 1379. 29357248 10.1021/acs.nanolett.7b05103

[296] E. J. Romans , E. J. Osley , L. Young , P. A. Warburton , W. Li , Appl. Phys. Lett. 2010, 97.

[297] A. Cui , W. Li , T. H. Shen , Y. Yao , J. C. Fenton , Y. Peng , Z. Liu , J. Zhang , C. Gu , Sci. Rep. 2013, 3, 2429. 23938336 10.1038/srep02429 PMC3741620

[298] R. Córdoba , D. Mailly , R. O. Rezaev , E. I. Smirnova , O. G. Schmidt , V. M. Fomin , U. Zeitler , I. Guillamón , H. Suderow , J. M. De Teresa , Nano Lett. 2019, 19, 8597. 31730351 10.1021/acs.nanolett.9b03153 PMC7005939

[299] I. Utke , P. Hoffmann , J. Melngailis , J. Vacuum Sci. Technol. B: Microelectron. Nanometer Struct. Process., Meas., and Phenom. 2008, 26, 1197.

[300] A. Yasaka , F. Aramaki , T. Kozakai , O. Matsuda , Hitachi Rev 2016, 65, 233.

[301] T. J. Blom , T. W. Mechielsen , R. Fermin , M. B. S. Hesselberth , J. Aarts , K. Lahabi , ACS Nano 2021, 15, 322. 33231428 10.1021/acsnano.0c03656 PMC7844821

[302] W. Tiddi , A. Elsukova , H. T. Le , P. Liu , M. Beleggia , A. Han , Nano Lett. 2017, 17, 7886. 29156134 10.1021/acs.nanolett.7b04190

[303] W. Tiddi , A. Elsukova , M. Beleggia , A. Han , Microelectron. Eng. 2018, 192, 38.

[304] J. A. Lewis , G. M. Gratson , Mater. Today 2004, 7, 32.

[305] E. J. Brandon , E. E. Wesseling , C. Vincent , W. B. Kuhn , IEEE Trans. Compon. Packaging Technol. 2003, 26, 517.

[306] T. Zhang , X. Wang , T. Li , Q. Guo , J. Yang , J. Mater. Chem. C 2014, 2, 286.

[307] X. Wang , L. Xu , G. Zheng , W. Cheng , D. Sun , Sci. China Technol. Sci. 2012, 55, 1603.

[308] R. R. Sarreal , P. Bhatti , Sensors 2020, 20, 6087. 33114773 10.3390/s20216087 PMC7663185

[309] D. A. Pardo , G. E. Jabbour , N. Peyghambarian , Adv. Mater. 2000, 12, 1249.

[310] Y. Wang , Y. Shi , C. X. Zhao , J. I. Wong , X. W. Sun , H. Y. Yang , Nanotechnology 2014, 25, 094010. 24522166 10.1088/0957-4484/25/9/094010

[311] A. Pepłowski , S. Rathi , B. Piotrkowski , R. Ziółkowski , D. Janczak , J. Krzemiński , M. Brosch , M. Jakubowska , Front. Neurosci. 2020, 14. 10.3389/fnins.2020.594235 PMC7658312 33192280

[312] H. G. Gnanasambanthan , D. Maji , ACS Appl. Electron. Mater. 2023, 5, 4426.

[313] A. Han , J. Chervinsky , D. Branton , J. A. Golovchenko , Rev. Sci. Instrum. 2011, 82. 10.1063/1.3601005 PMC3144963 21721733

[314] G. McKerricher , M. Vaseem , A. Shamim , Microsyst. Nanoeng. 2017, 3, 16075. 31057848 10.1038/micronano.2016.75 PMC6444987

[315] E. Saleh , F. Zhang , Y. He , J. Vaithilingam , J. L. Fernandez , R. Wildman , I. Ashcroft , R. Hague , P. Dickens , C. Tuck , Adv. Mater. Technol. 2017, 2, 1700134.

[316] 3D Printing and Additive Manufacturing 2019, 6, 165. 10.1089/3dp.2018.0175 PMC6594419 31860224

[317] J. A. Rogers , R. J. Jackman , G. M. Whitesides , D. L. Olson , J. V. Sweedler , Appl. Phys. Lett. 1997, 70, 2464.

[318] A. Imai , S. Takahashi , S. Furubayashi , Y. Mizuno , M. Sonoda , T. Miyazaki , E. Miyashita , T. Fujie , Adv. Mater. Technol. 2023, 8, 2300300.

[319] T. Yousefi , M. Taghadosi , A. Dabbaghian , R. Siu , G. Grau , G. Zoidl , H. Kassiri , IEEE Trans. Biomed. Circuits Syst. 2020, 14, 1274. 32976106 10.1109/TBCAS.2020.3026937

[320] A. Khalifa , Johns Hopkins University 2019.

[321] J.‐U. Park , M. Hardy , S. J. Kang , K. Barton , K. Adair , D. k. Mukhopadhyay , C. Y. Lee , M. S. Strano , A. G. Alleyne , J. G. Georgiadis , P. M. Ferreira , J. A. Rogers , Nat. Mater. 2007, 6, 782. 17676047 10.1038/nmat1974

[322] G. L. Goh , V. Dikshit , R. Koneru , Z. K. Peh , W. Lu , G. D. Goh , W. Y. Yeong , Int. J. Adv. Manuf. Technol. 2022, 120, 2573.

[323] M. S. Onses , E. Sutanto , P. M. Ferreira , A. G. Alleyne , J. A. Rogers , Small 2015, 11, 4237. 26122917 10.1002/smll.201500593

[324] O. A. Basaran , Am. Inst. Chem. Eng. AIChE J. 2002, 48, 1842.

[325] P. Galliker , J. Schneider , H. Eghlidi , S. Kress , V. Sandoghdar , D. Poulikakos , Nat. Commun. 2012, 3, 890. 22692533 10.1038/ncomms1891

[326] S. J. P. Kress , P. Richner , S. V. Jayanti , P. Galliker , D. K. Kim , D. Poulikakos , D. J. Norris , Nano Lett. 2014, 14, 5827. 25180812 10.1021/nl5026997

[327] P. Galliker , J. Schneider , L. Rüthemann , D. Poulikakos , Proc. Natl. Acad. Sci. USA 2013, 110, 13255. 23898173 10.1073/pnas.1305886110 PMC3746916

[328] J. Schneider , P. Rohner , D. Thureja , M. Schmid , P. Galliker , D. Poulikakos , Adv. Funct. Mater. 2016, 26, 833.

[329] P. Richner , P. Galliker , T. Lendenmann , S. J. P. Kress , D. K. Kim , D. J. Norris , D. Poulikakos , ACS Photonics 2016, 3, 754.

[330] D.‐Y. Lee , Y.‐S. Shin , S.‐E. Park , T.‐U. Yu , J. Hwang , Appl. Phys. Lett. 2007, 90, 081905.

[331] D. Gao , J. G. Zhou , Int. J. Bioprint. 2019, 5. 10.18063/ijb.v5i1.166 PMC7415859 32782978

[332] H. Zhou , Y. Song , Adv. Electron. Mater. 2022, 8, 2200728.

[333] W. Chen , C. S. Fernandez , L. Xu , E. Velliou , S. Homer‐Vanniasinkam , M. K. Tiwari , in 3D Printing in Medicine (Ed: D. M. Kalaskar ), Woodhead Publishing, Duxford, UK 2023.

[334] J. Kim , H. Oh , S. S. Kim , J. Aerosol Sci. 2008, 39, 819.

[335] I. Woods , D. Spurling , S. Sunil , J. Maughan , J. Guttierez‐Gonzales , A. Dervan , V. Nicolosi , F. J. O'Brien , bioRxiv 2024, 2024.

[336] O. A. Selim , S. Lakhani , S. Midha , A. Mosahebi , D. M. Kalaskar , Tissue Eng., Part B 2021, 28, 295. 10.1089/ten.TEB.2020.0355 33593147

[337] A. Zennifer , D. R. Chellappan , P. Chinnaswamy , A. Subramanian , D. Sundaramurthi , S. Sethuraman , Biofabrication 2024, 16, 045015. 10.1088/1758-5090/ad5fbe 38968935

[338] M. Alhendi , F. Alshatnawi , E. M. Abbara , R. Sivasubramony , G. Khinda , A. I. Umar , P. Borgesen , M. D. Poliks , D. Shaddock , C. Hoel , N. Stoffel , T.‐K. H. Lam , Addit. Manuf. 2022, 54, 102709.

[339] E. B. Secor , Flexible Printed Electron. 2018, 3, 035002.

[340] C. Hollar , Z. Lin , M. Kongara , T. Varghese , C. Karthik , J. Schimpf , J. Eixenberger , P. H. Davis , Y. Wu , X. Duan , Y. Zhang , D. Estrada , Adv. Mater. Technol. 2020, 5, 2000600. 33738334 10.1002/admt.202000600 PMC7968868

[341] T. Pandhi , C. Cornwell , K. Fujimoto , P. Barnes , J. Cox , H. Xiong , P. H. Davis , H. Subbaraman , J. E. Koehne , D. Estrada , RSC Adv. 2020, 10, 38205. 35517530 10.1039/d0ra04786d PMC9057201

[342] M. T. Craton , J. D. Albrecht , P. Chahal , J. Papapolymerou , IEEE Trans. Compon., Packag., Manuf. Technol. 2021, 11, 865.

[343] Y. Gu , D. Park , S. Gonya , J. Jendrisak , S. Das , D. R. Hines , Addit. Manuf. 2019, 30, 100843.

[344] L.‐k. Tsui , Y. Sui , T. M. Hartmann , J. Dye , J. M. Lavin , *ECS Meeting Abstr*. 2023, *MA2023‐01*, 1558.

[345] S. Krainer , C. Smit , U. Hirn , RSC Adv. 2019, 9, 31708. 35527935 10.1039/c9ra04993b PMC9072721

[346] B. Derby , Annu. Rev. Mater. Res. 2010, 40, 395.

[347] G. Grau , J. Cen , H. Kang , R. Kitsomboonloha , W. J. Scheideler , V. Subramanian , Flexible Print. Electron. 2016, 1, 023002.

[348] R. Kitsomboonloha , S. Morris , X. Rong , V. Subramanian , Langmuir 2012, 28, 16711. 23110647 10.1021/la3037132

[349] R. Wu , J. K. O. Sin , IEEE Electron Device Lett. 2011, 32, 60.

[350] H. T. Le , I. Mizushima , Y. Nour , P. T. Tang , A. Knott , Z. Ouyang , F. Jensen , A. Han , Microsyst. Nanoeng. 2018, 4, 17082.

[351] H. T. Le , Y. Nour , Z. Pavlovic , C. O. Mathúna , A. Knott , F. Jensen , A. Han , S. Kulkarni , Z. Ouyang , IEEE Trans. Power Electron. 2019, 34, 74.

[352] H. Y. Li , L. Xie , L. G. Ong , A. Baram , I. Herer , A. Hirshberg , S. C. Chong , S. Gao , D. L. Kwong , IEEE Electron Device Lett. 2012, 33, 432.

[353] T. Sun , H. Sharma , P. M. Raj , F. Yoshihiro , S. Hachiya , K. Takemura , R. Tummala , IEEE Trans. Compon., Packag., Manuf. Technol. 2020, 10, 134.

[354] S. J. Bleiker , A. C. Fischer , U. Shah , N. Somjit , T. Haraldsson , N. Roxhed , J. Oberhammer , G. Stemme , F. Niklaus , IEEE Trans. Compon., Packag., Manuf. Technol. 2015, 5, 21.

[355] H. Li , J. Liu , T. Xu , J. Xia , X. Tan , Z. Tao , Micromachines 2018, 9. 10.3390/mi9100528 PMC6215278 30424461

[356] W. Zhang , J. Gu , G. Xu , L. Luo , X. Li , Microelectron. J. 2020, 101, 104798.

[357] Z. Tao , J. Sun , H. Li , Y. Huang , H. Li , T. Xu , H. Wu , IEEE Electron Device Lett. 2020, 41, 1090.

[358] R. Anthony , E. Laforge , D. P. Casey , J. F. Rohan , C. O'Mathuna , J. Micromech. Microeng. 2016, 26, 105012.

[359] B. Magali , O. D. Terence , O. B. Joe , M. Paul , M. Seán Cian Ó , J. Micromech. Microeng. 2002, 12, 444.

[360] D. Bourrier , A. Ghannam , M. Dilhan , H. Granier , Microsyst. Technol. 2014, 20, 2089.

[361] D. V. Harburg , A. J. Hanson , J. Qiu , B. A. Reese , J. D. Ranson , D. M. Otten , C. G. Levey , C. R. Sullivan , IEEE J. Emerg. Select. Top. Power Electron. 2018, 6, 1280.

[362] R. Anthony , N. Wang , D. P. Casey , C. Ó Mathúna , J. F. Rohan , J. Magn. Magn. Mater. 2016, 406, 89.

[363] M. Colella , D. Z. Press , R. M. Laher , C. E. McIlduff , S. B. Rutkove , A. M. Cassarà , F. Apollonio , A. Pascual‐Leone , M. Liberti , G. Bonmassar , Med. Phys. 2023, 50, 1779. 36502488 10.1002/mp.16148 PMC10033376

[364] X. Yu , W. Huang , M. Li , T. M. Comberiate , S. Gong , J. E. Schutt‐Aine , X. Li , Sci. Rep. 2015, 5, 9661. 25913217 10.1038/srep09661 PMC5386192

[365] W. Huang , Z. Yang , M. D. Kraman , Q. Wang , Z. Ou , M. M. Rojo , A. S. Yalamarthy , V. Chen , F. Lian , J. H. Ni , S. Liu , H. Yu , L. Sang , J. Michaels , D. J. Sievers , J. G. Eden , P. V. Braun , Q. Chen , S. Gong , D. G. Senesky , E. Pop , X. Li , Sci. Adv. 2020, 6, eaay4508. 32010770 10.1126/sciadv.aay4508 PMC6968933

[366] W. Huang , J. Zhou , P. J. Froeter , K. Walsh , S. Liu , M. D. Kraman , M. Li , J. A. Michaels , D. J. Sievers , S. Gong , X. Li , Nat. Electron. 2018, 1, 305.

